# Expression and Functional Analysis of the Propamocarb-Related Gene *CsMCF* in Cucumber

**DOI:** 10.3389/fpls.2019.00871

**Published:** 2019-07-04

**Authors:** Fan Zhang, Ming Xin, Siqi Yu, Dong Liu, Xiuyan Zhou, Zhiwei Qin

**Affiliations:** College of Horticulture and Landscape Architecture, Key Laboratory of Biology and Genetic Improvement of Horticultural Crops (Northeast Region), Northeast Agricultural University, Harbin, China

**Keywords:** cucumber, propamocarb residue, propamocarb, *CsMCF*, functional analysis

## Abstract

Propamocarb (PM), a carbamate fungicide, can effectively control downy mildew on cucumber. However, due to the large-scale and high-dose use of this fungicide, PM residues have become a major problem in cucumber production. In this report, the cucumber cultivar “D0351” (with the lowest residual PM content) and the cucumber cultivar “D9320” (with the highest residual PM content) were used as experimental materials. The candidate gene *CsMCF*, which is related to a low residual PM content in cucumber, was screened by high-throughput tag-sequencing (Tag-Seq) and PM analysis, and its role in reducing PM residue in cucumber was explored. *CsMCF* was cloned and obtained. This gene contains an open reading frame of 1026 bp, encodes 341 amino acids and contains 3 Mito-carr domains. The encoded protein is a hydrophobic protein with 4 distinct transmembrane structures but no signal peptide cleavage sites. The subcellular localization of the protein is the cytoplasm. Evolutionary tree analysis showed that *CsMCF* had the highest homology to a gene from the melon *Cucumis melo* L. (XM_008464998.2). The core elements of the promoter include *cis-*acting elements, such as those related to salicylic acid (SA), jasmonic acid (JA), gibberellin (GA), and abscisic acid (ABA). Following PM treatment, *CsMCF* was significantly upregulated at most time points in different parts of the fruit, leaf, stem and root of “D0351,” while expression was downregulated at most time points in the fruit, leaf and stem of “D9320.” The order of the expression levels in different cucumber organs was as follows: fruit>leaf > stem > root. *CsMCF* was specifically expressed in the stems and leaves of “D0351.” The PM residues in *CsMCF* (+)-overexpressing T0 and T1 cucumber fruits were significantly lower than those in the wild type, while the PM residues in *CsMCF* (-)-overexpressing T0 and T1 cucumber fruits were significantly higher. The qRT-PCR results showed that *CsMCF* can respond to biotic and abiotic stresses, actively respond to PM treatment and play a role in reducing PM residues in cucumber fruits.

## Introduction

Cucumber is one of the main vegetable crops grown under protected cultivation in China. This vegetable is very popular because of its crispness, tenderness, fragrance and nutritional value. Continuous cropping and annual production have increased the susceptibility of cucumber to downy mildew, leading to chlorosis of the leaves, reducing the photosynthetic efficiency of the leaves and the accumulation of organic matter in cucumber, thereby seriously affecting the yield and quality of cucumber. The use of the fungicide propamocarb (PM) is one of the fastest and most common methods to control downy mildew of cucumber (Zhang et al., 2013). However, the potential safety hazards and environmental pollution caused by fungicides should not be underestimated, and these issues should be further investigated. Studies have shown that human consumption of vegetables with excessive fungicides can lead to dizziness, sleepiness, loss of appetite, liver damage and a series of acute and chronic poisoning symptoms; fungicides are toxic and can accumulate in large quantities in the human body, leading to fetal malformation. Several animal experiments have also proven that fungicides residues have carcinogenic effects. Therefore, addressing the issue of fungicides residues in cucumber is urgently needed. Botanical morphological characteristics have been clarified in the established evaluation index system of pesticide residues in cucumber germplasm resources. Low and high PM residues of deltamethrin, PM and myclobutanil have been identified in cucumber varieties according to this system (Liu, 2010). The genetic characteristics related to cucumber PM residues have been analyzed in a previous study. The researchers suggested that the PM residues in cucumber fruits were a quantitative trait controlled by multiple genes, and a PM residue-related QTL was detected (Ma, 2010). Given the fluidity and variation in PM residues in cucumber, the residual quantity and variation rate of low-PM-residue varieties have been shown to be lower than those of high-PM-residue varieties (Zhao, 2013). Under PM stress, differentially expressed genes in cucumber fruits were screened by Solexa and comparative genomics methods on the basis of physiological and biochemical studies of low-PM-residue cucumbers (Wu et al., 2013). According to research on the genes responding to PM stress in cucumber varieties with different levels of PM residues [*CsABC19* (Meng et al., 2016), *CsWRKY30* (Li et al., 2016), *CsSDH* (Guo, 2013) and *CsDIR16* (Liu et al., 2018)], the resistance of transgenic *Arabidopsis thaliana* or cucumber to PM stress can be improved significantly by the genes mentioned above.

*CsMCF* contains the Mito-carr domain and belongs to mitochondrial carrier family (MCF) proteins. This protein can directly control the entry and exit of metabolites in the mitochondrial inner membrane (Palmieri, 2004). In the mitochondrial vector family, specific protein vectors distribute coenzyme factors such as NAD and CoA to the corresponding cell chambers. These coenzyme factors can be utilized by many enzymes and are indispensable substrates for various enzymatic reactions in cells. In NCBI, *CsMCF* is defined as a *Cucumis sativus* peroxisomal nicotinamide adenine dinucleotide carrier, also known as peroxisome NAD vector, which is a type of peroxisome NAD transporter protein in plants. This protein is primarily involved in facilitating NAD transport, promoting substance conversion between NAD and NADH and catalyzing the transport of CoA (Houtkooper et al., 2010). NAD is a ubiquitous biological molecule that has a role in many basic physiological processes of living cells (Pollak et al., 2007; Houtkooper et al., 2010). Pathogenic analysis revealed that the fungicide PM (carbamate) can inhibit the decomposition of acetylcholinesterase, which leads to the accumulation of acetylcholine (acetylcholine is a neurotransmitter) in the body, affecting the normal nerve conduction process in organisms and even leading to the poisoning and death of organisms. The peroxisome NAD vector plays an important role in the treatment of neurological diseases. Because it presents detoxification metabolism, peroxisome NAD vector is widely used in the prevention of type 2 diabetes mellitus. In addition to neurological diseases and type 2 diabetes mellitus, several other pathological conditions are also closely related to NAD, such as leukemia. At present, the study of peroxisome NAD vector in cucumber has not been reported. The function of the peroxisome NAD vector in cucumber has been identified, and its roles in metabolic pathways are understood. The elucidation of unknown regulatory mechanisms may help gain a better understanding of the physiological role of MCF in cell metabolism. Based on the results of Solexa high-throughput sequencing (Wu et al., 2013), a candidate gene, Cucsa.368700.1, was shown to reduce the PM residue. This gene belongs to the mitochondrial carrier superfamily and was named *CsMCF*. Quantitative fluorescence analysis showed that the gene responded significantly to PM treatment in the low-PM-residue cucumber cultivar “D0351.” However, the response mechanism of *CsMCF* following PM treatment of cucumber has not been reported. *CsMCF* was cloned from “D0351” fruit cDNA, and its sequence domains and gene structure were analyzed. The physical and chemical properties of the protein were explored through bioinformatics analysis, the homologous sequences were compared, and subcellular localization analysis was carried out. Changes in *CsMCF* expression in high- and low-residue lines exposed to PM were assessed, and relative analysis of residues in transgenic cucumber was performed. The purpose of this study was to explore whether *CsMCF* could actively respond to the PM treatment and play a role in reducing PM residues in cucumber to lay a foundation for the study of the molecular mechanism of low PM residues in cucumber and the cultivation of new varieties with low agricultural residues.

## Materials and Methods

### Experimental Materials

The homozygous cucumber lines “D0351” (with low residual PM contents) and “D9320” (with high residual PM contents) were selected as experimental materials (Liu, 2010). The seeds were provided by the cucumber research group of Northeast Agricultural University, Harbin, China.

### Treatment of Test Materials

Conventional barrel-and-frame cultivation methods were adopted for unified management. When the cucumber cultivars “D0351” and “D9320” was ripe at the 10th node, the plants were sprayed with 400x PM solution, and the control group was sprayed with the same amount of distilled water until the surface of the leaves and fruits began to drip. Samples were taken at 1, 6, 12, 24, 48, and 72 h after treatment, frozen in liquid nitrogen and stored at -80°C for the determination of gene expression. The experiment was repeated three times.

Overexpression (OE), antisense expression (AS) vectors and an empty vector were constructed for the genetic transformation of the “D0351” and “D9320” cucumber cultivars. In total, 13 OE lines, 20 AS lines and 4 empty vector lines were obtained in the “D0351” T_0_ generation. The three OE lines with the highest expression of *CsMCF* (lines 2, 9, and 10) and the AS lines with the lowest expression (lines 4, 5, and 15) were selected for further study. A total of 12 OE lines, 9 AS lines and 4 empty vector lines were obtained in the “D9320” T_0_ generation. The three OE lines with the highest expression of *CsMCF* were lines 1, 3, and 4, and the AS lines with the lowest expression were lines 2, 4, and 7. These OE and AS lines were planted in a greenhouse self-pollination to obtain T_1_ generation seeds. The propamocarb treatment of transgenic plants is the same as above. These samples were used for the determination of PM residues. The functional leaves were cut at days 0, 2, 4, 6, 8, and 10, and the physiological indexes were determined after mixing. The experiment was repeated three times.

“D0351” wild-type plants, “D9320” wild-type plants and transgenic T_1_ generation plants were planted in soil at the Biotron in March 2016 under the following conditions: 28/18°C (12 h day/12 h night) and 70% relative humidity. Three true-leaf seedlings were used to determine changes in *CsMCF* expression after treatment with salicylic acid (SA), jasmonic acid (JA), gibberellin (GA), abscisic acid (ABA), PEG4000, cold, NaCl and *Corynespora cassiicola* Wei (Cor) stress [SA-induced treatment: foliar spraying with 0.5 mmol/L SA (Wu et al., 2009); JA-induced treatment: foliar spraying with 100 μmol/L JA (Liu et al., 2018); GA-induced treatment: foliar spraying with 100 μmol/L GA; ABA-induced treatment: foliar spraying with 100 μmol/L ABA; PEG (drought stress): seedlings were irrigated with 50 mL of 40% PEG4000, and leaves were harvested 8 days after treatment; cold treatment (low temperature stress): the day and night temperatures were set to 10 ± 0.5°C/5 ± 0.5°C, the illumination time was 16 h, the light intensity was 4000 lx, and the low temperature stress period was 10 days; NaCl (high salt stress): each plant was irrigated with 50 mL of 400 mmol/L NaCl, treated once every 3 days, and sampled on the 8th day; *C. cassiicola* Wei (disease stress): seedlings were sprayed with 1 × 10^5^ colony-forming units/mL Cor, and leaves were harvested 24 h after treatment]. Five plants with identical growth were selected as replicates for each treatment, and leaves were harvested 24 h after treatment. After liquid nitrogen freezing, they were stored at -80°C for analysis of expression patterns induced by external factors.

### Cloning and Bioinformatics Analysis of *CsMCF*

The full-length coding sequence (CDS) of *CsMCF* was obtained by BLAST alignment with the cucumber genome database^1^. Primer Premier 5.0 software was used to design primers for the full-length coding region of the cloned gene. *CsMCF-*F: 5′-CTTCTTTGCTTTCTCTAATTCGG-3′, *CsMCF-*R: 5′-AATGGGGTCATATAAGCATTTTATC-3′. With “D0351” cDNA as a template, PCR amplification was performed as follows: 94°C for 5 min, 94°C for 30 s, 56.5°C for 30 s, 72°C for 30 s, 35 cycles, 72°C for 10 min. The amplified PCR products were detected by agarose gel electrophoresis, and a colloid recovery kit (TransGen Biotech) was used to recover the target bands. The recovered fragments were attached to the T3 vector (TransGen Biotech). After the reaction, the sample was transformed into *Escherichia coli* DH5α competent cells by thermal shock, followed by overnight incubation on LB agar plates containing X-Gal, IPTG and Amp at 37°C. Single white bacterial colonies were isolated and sent to be sequenced (GENEWIZ).

The *CsMCF* protein sequence was analyzed using the Conserved Domain Database (CDD) of NCBI^2^, and ProtParam^3^ was used to assess the physicochemical properties of the amino acids. The ProtScale website^4^ was used to predict the hydrophilicity of gene-encoded proteins. Online prediction of the protein transmembrane structure was conducted using the TMHMM server v.2.0. Signal peptide prediction of cucumber proteins was performed using SignalP 4.1 Server^5^, and prediction of protein secondary structure was performed using the SPOMA online tool^6^. The phylogenetic tree was constructed by the neighbor-joining method in MEGA software.

### Analysis of *CsMCF* Expression Patterns

qRT-PCR analysis of total RNA extracted from 100 mg of fresh cucumber using TRIzol (Invitrogen^TM^) was performed after addition of liquid nitrogen into a sterilized grinding bowl, and the purity of the RNA was detected by gel electrophoresis. A reverse transcription kit (Toyobo, Japan) was used for reverse transcription of RNA into single-stranded cDNA, and the products were stored at -20°C for further analysis.

Primers were designed with the online software https://pga.mgh.harvard.edu/primerbank/. *CsMCF*-qF: GAGCGCTGACTCCACTAGAT, *CsMCF*-qR: AGAAGGGTTGCTGACCATGA. Relative quantitation of gene expression was performed using *CsEF1α* (Wan et al., 2010) (GenBank accession number: XM_004138916) as a control (CsEF1a-qrF: CCAAGGCAAGGTACGAT GAAA, CsEF1a-qrR: AGAGATGGGAACGAAGGGGAT). Three biological replicates and three technical replicates were performed. The 2-ΔΔCT method (Schmittgen and Livak, 2008) was used to analyze the real-time qRT-PCR results.

### Sequence Analysis of the *CsMCF* Promoter

The 2000 bp promoter region upstream of *CsMCF* was obtained from the cucumber gene database (see footnote 1), and the *cis-*acting elements in the promoter region were analyzed using the online tool PlantCARE^7^ (Lescot et al., 2002).

### Subcellular Localization of *CsMCF*

Construction of the fusion expression vector of *CsMCF* and GFP was performed. Primers were designed with *Hind*III and *BamH*I restriction sites (*CsMCF*-LF: 5′-GCTCTAGAATGGCCGCAATCCAGCTTCTC-3′, *CsMCF*-LR: 5′-CGGGATCCTAATTGACGAAGAAGCTTAACACCG AAC-3′). The *CsMCF* open reading frame without a stop codon was amplified. The pEASY-T3-CsMCF vector was constructed and transformed into *E. coli* and identified for sequencing. The pEASY-T3-CsMCF instantaneous expression vector plasmid and pGII-eGFP vector plasmid were digested by fast endonuclease *Hind*III and *BamH*I. The purified product was recovered by gel electrophoresis and ligated using T4 ligase. The fusion expression vector 35S-CsMCF-eGFP was obtained and transformed into *E. coli* and identified. Protoplast extraction and transformation in *A. thaliana* were described in the literature (Yoo et al., 2007). Added 35S-CsMCF-eGFP plasmids (1 ug/ul 20 ul) to 1 ml Arabidopsis protoplasts (2 × 10^7^/ml). Laser confocal microscopy (TCS SP2 Leica, Germany) was used for observation. The fluorescence of GFP was observed at an excitation wavelength of 488 nm and an emission wavelength of 530 nm.

The fusion expression vector 35S-CsMCF-eGFP and mitochondrion marker was transformed into rice protoplasts. Protoplast extraction and transformation in rice were described in the literature (Li, 2013). Added 35S-CsMCF-eGFP plasmids (1 ug/ul 20 ul) to 200 ul rice protoplasts (2 × 10^5^/ml). Laser confocal microscopy (FV10-ASW) was used for observation. The fluorescence of GFP was observed at an excitation wavelength of 480 nm and an emission wavelength of 510 nm.

### Genetic Transformation of Cucumber by *CsMCF*

With full-length primers and CsMCF-F, CsMCF-R, and pEASY-T3-CsMCF plasmids as templates, the target gene was amplified by PCR. The vector PCXSN-1250 (Chen et al., 2009) was digested by *Xcm*I. T4 ligase linked the target fragment to the vector, and the vector was transformed into *E. coli*. The construct of pCXSN-CsMCF was confirmed by PCR and sequencing. The constructed expression vectors were transferred into *Agrobacterium tumefaciens* LBA4404 by the freeze-thaw method to infect cucumber cotyledon nodes. Genetic transformation technology of cucumber was used, and MS medium containing 1.0 mg/L glyphosate was screened and used for differentiation medium. After 20 days, cotyledon nodes began to differentiate, and differentiated buds grew. When differentiated buds grew up, they were cut off and transferred into rooting medium. After the main roots and fibrous roots grew, they were transplanted and domesticated in an incubator to obtain T_0_ transgenic cucumber (Zhang et al., 2014). The domesticated transgenic seedlings were planted in a greenhouse for unified management and self-pollination to obtain T_1_ generation seeds. The DNA of transgenic cucumber was extracted by the CTAB method, and the sequence of the pCXSN vector was used as the primer. The transgenic plants were identified by PCR and qPCR (both the T_0_ and T_1_ plants).

### Determination of Residual PM

Sample (12.5 g) was mixed with 25 mL of acetonitrile and homogenized with a high-speed homogenizer (Heidolph Silent Crusher-M^®^) for 2 min at 15000 × g and incubated at room temperature for 0.5 h. The homogeneous acetonitrile was extracted into a centrifuge tube containing 3 g NaCl, rotated on a vortex for 1 min and centrifuged for 5 min at 5000 × g. Five milliliters of each supernatant was dried with a sample concentrator (Termovap) at 60°C, and 2.5 mL acetone was added. The mixture was then filtered through a 0.22 μm polypropylene filter. After the filtration membrane solution was clarified, the filter membrane solution stood for several minutes to observe the presence of turbidity; if particles were present, the solution was passed through the membrane again until no particles remained (NY/T761-2008, 2008). An Agilent 7890B-5977A gas chromatography system (Agilent Technologies) equipped with a capillary column (HP-5MS, 30 m × 0.25 mm × 0.25 μm) was used to analyze the level of PM residues. The parameters were as follows: programmed temperature: maintained for 5 min at 70°C, 150°C at 25°C/min, 200°C at 3°C/min, and 280°C at 20°C/min for 10 min; sample inlet: 250°C, non-split injection, flow rate of 1.0 mL/min, transmission line 250°C, four-stage rod 150°C, ion source 230°C (Liu et al., 2018).

### Detection of Physiological and Biochemical Indexes

The 0.5 g fresh sample of cucumber leaves was selected, added 3 ml phosphate buffer (PH = 7.8) with ice bath grinding centrifuged for 20 min at 10500 rpm. They were used as reaction liquid and stored at 4°C for detection of physiological and biochemical indexes.

POD was performed as guaiacol method (Bradford, 1976)with minor modification. 112 uL guaiacol was added into 200 ml phosphate buffer (PH = 6.0), heated until it dissolves sufficiently, then added 19 uL H_2_O_2_ (30%). The obtained solution were used as reaction mixture and stored at 4°C. 20 uL reaction mixtures was added into 3 ml reaction liquid, and 20 uL phosphate buffer was added into 3 ml reaction liquid as control. The samples were put into an ultraviolet–visible spectrophotometer (Shimadzu, Japan) and recorded the OD value at 470 nm/min (OD values for 0, 1, 2, and 3 min).

SOD was performed as NBT method (Aebi, 1984) with minor modification. Reaction mixture were prepared by H_2_O, phosphate buffer, Met, NBT, EDTA-Na2 and lactochrome in proportion of 5:30:6:6:6:6.50 uL reaction mixture was added into 3 ml reaction liquid. 50 uL phosphate buffer was added into 3 ml reaction liquid as control group. The control group was divided into two groups, control group 1 was treated in dark and control group 2 reacted in normal light, determination of absorbance with the ultraviolet–visible spectrophotometer (Shimadzu, Japan) at OD560.

CAT was performed as H_2_O_2_ method (Giannakoula et al., 2010) with minor modification. 50 ml H_2_O_2_ (0.1 mol/L) was added into 200 ml phosphate buffer (PH = 7.0). The obtained solution were used as reaction mixture and stored at 4°C. 100 uL reaction mixtures was added into 3 ml reaction liquid. 100 uL phosphate buffers was added into 3 ml reaction liquid as control group. The samples were put into an ultraviolet–visible spectrophotometer (Shimadzu, Japan) and recorded the OD value at 240 nm/min (OD values for 0, 1, 2, and 3 min).

MDA was detected as described in a previous study (Foyer and Halliwell, 1976). 2 mL TBA (0.67%) was added into 1 ml reaction liquid. 2 mL TBA (0.67%) was added into 1 ml distilled water as control group. Sealed the orifice of the test tube to avoid liquid volatilization. After 15 min of boiling water bathed, it was cooled and introduced into the centrifugal tube. Centrifuged for 20 min at 4000 rpm, then detected at 600, 532, and 450 nm with the ultraviolet–visible spectrophotometer (Shimadzu, Japan).

### Statistical Analysis

Three biological replicates and three technical replicates were performed. The data were subjected to statistical analyses using the data processing system Origin8.0 and DPS 9.5. Data were expressed as the mean ± SD. Significant differences between the treatment and control groups were confirmed by Student’s *t*-tests. The data were analyzed by analysis of variance (*p* < 0.05 is significance level and *p* < 0.001 is extremely significant level).

## Results

### Cloning and Bioinformatics Analysis of *CsMCF*

Using CsMCF-F and CsMCF-R as primers and cucumber “D0351” fruit DNA as a template, we amplified a band of approximately 1000 bp by PCR (Supplementary Figure S1A). Recovery and purification of the target fragment was performed, and the product was cloned into the pEASY-T3 vector. Then, the plasmid was extracted and verified by restriction enzyme digestion (Supplementary Figure S1B). After sequencing, the base sequence was shown to be identical to that of the coding region of a gene in the cucumber genome database (Csa5G198760.1). The total length of the base sequence was 1026 bp, indicating that the target gene had been successfully obtained.

The CsMCF protein contains 341 amino acids and has a predicted theoretical molecular weight of 37.225 kDa, a theoretical isoelectric point of 9.84, an atomic composition of C_1686_H_2734_N_452_O_467_S_13_, a total number of atoms of 5352, and a fat coefficient of 100.91. An NCBI BLAST analysis showed that the protein had the Mito-carr domain (Supplementary Figure S2A); thus, the gene encoding the protein was named *CsMCF*.

The prediction of CsMCF protein transmembrane regions showed that there were four distinct transmembrane regions (Supplementary Figure S2B). The protein encoded by the gene did not show any significant signal peptide cleavage sites (Supplementary Figure S2C), indicating that the protein may be localized to the cytoplasmic matrix or organelle matrix and is not a membrane protein or secretory protein. The highest hydrophobic value was 2.900 at amino acid 311th, and the maximum hydrophilic value was -3.111 at the 40th amino acid position. The average hydrophilic coefficient was 0.076, indicating a hydrophobic protein (Supplementary Figure S2D).

The secondary structure of the protein was analyzed (Supplementary Figure S2F). The results showed that there were 179 alpha helixes, accounting for 52.49% of the entire polypeptide chain; 36 extended main chains, accounting for 10.56% of the entire polypeptide chain; 18 beta helixes, accounting for 5.28% of the entire polypeptide chain; and 108 random coils, accounting for 31.67% of the entire polypeptide chain. The whole protein sequence was analyzed by the protein homology program Phyre to predict the tertiary structure of the protein (Supplementary Figure S2E).

### Phylogenetic Tree of *CsMCF*

**FIGURE 1:**
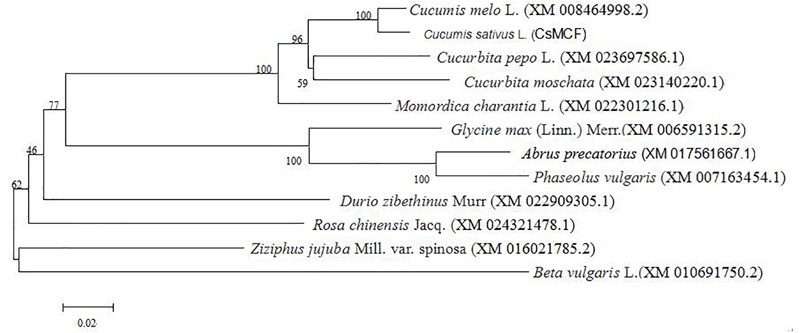
Phylogenetic tree of *CsMCF.* The amino sequences were subjected to phylogenetic analysis using the neighbor-joining method in MEGA (version 5.2) software. All sequences data are shown in Supplementary Table S1.

The evolutionary tree of the amino acid sequence was constructed using MEGA 5.0 software to explore the evolutionary relationship between *CsMCF* and other plant mitochondrial vector genes. As shown in Figure 1, the relationship between cucumber *CsMCF* and a gene from the melon *Cucumis melo* L. (XM_008464998.2) was the closest, with 97.56% homology. The *CsMCF* gene may be involved in biochemical reactions similar to that in melon. The homology with sugar beet was only 73%. The evolutionary tree showed that plants of the same family or group were clustered into one group, but plants of different families and genera also had high homology, indicating that members of the Mito-carr superfamily are highly conserved in the evolutionary process.

### Analysis of Promoter *cis*-Acting Elements

The 2000 bp promoter sequence upstream of the open reading frame of *CsMCF* was extracted from the gene database. The promoter sequence of *CsMCF* (Table 1) contains basic elements that regulate eukaryotic gene transcription, such as CAAT and the G-box, which may constitute the core sequence of the *CsMCF* gene promoter. This sequence also has *cis-*acting elements related to ABA induction (ABRE), methyl jasmonate (CGTCA motif), GAs (two GARE motifs), SA (TCA element), and growth hormones (two TGA elements); four binding sites for the stress-related protein family MYB; and one MYB recognition site. Thus, *CsMCF* expression may be regulated by several hormones and stress-related regulation.

**Table 1:** Distribution and function of *cis-*acting elements in *CsMCF* promoter.

Name	Sequence	Function	Position
ABRE	ACGTG	*Cis-*acting elements in abscisic acid reaction	665
CCAAT-box	CAACGG	MYBHV1 binding site	994
CGTCA-motif	CGTCA	*Cis-*acting regulatory elements involved in the reaction of methyl jasmonate	90
G-box	CACGTC	*Cis-*acting regulatory element of light response	664
GARE-motif	TCTGTTG	Gibberellin response element	144, 321
TCA-element	TCAGAAGAGG	*Cis-*acting elements for salicylic acid reaction	69
TGA-element	AACGAC	Auxin response element	498, 661
Myb-binding site	CAACAG	MYB binding site	144, 378, 322, 490
MYB recognition site	CCGTTG	MYB recognition site	994

### Subcellular Localization of the *CsMCF* Protein

**FIGURE 2:**
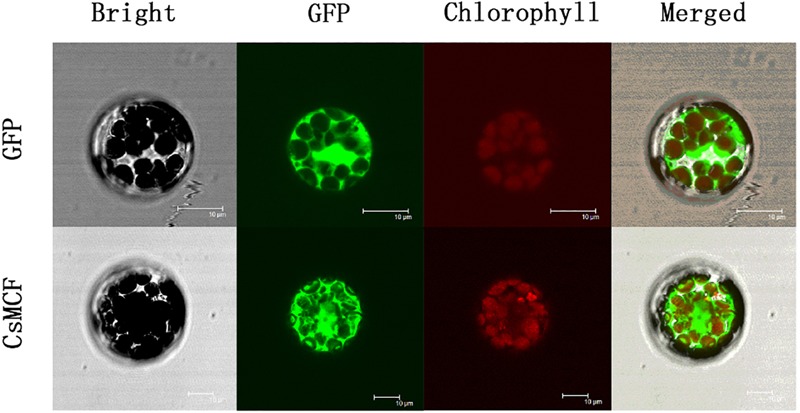
Subcellular localization of CsMCF-pGII-EGFP protein in Arabidopsis protoplasts. Subcellular localization of the CsMCF-pGII-EGFP fusion protein in Arabidopsis protoplasts. Images show protoplasts prepared from 3- to 4-week-old Arabidopsis leaves expressing CsMCF-pGII-EGFP (bottom row) or pGII-EGFP (upper row). Bright-field illumination, GFP fluorescence, chlorophyll fluorescence, and an overlay of GFP and chlorophyll fluorescence are shown. Scale bars, 10 μm.

To determine the location of the CsMCF protein in cells, we introduced the fusion expression vectors CsMCF-GFP and GFP empty vectors into *A. thaliana* protoplasts for fluorescence signal detection. As shown in Figure 2, green fluorescence was observed in the whole protoplast of the GFP empty vector control group. The green fluorescence was enriched in the cytoplasm of the cells transfected with the CsMCF-GFP fusion expression vector. Thus, we concluded that *CsMCF* is located in the cytoplasm and is a cytoplasmic protein.

**FIGURE 3:**
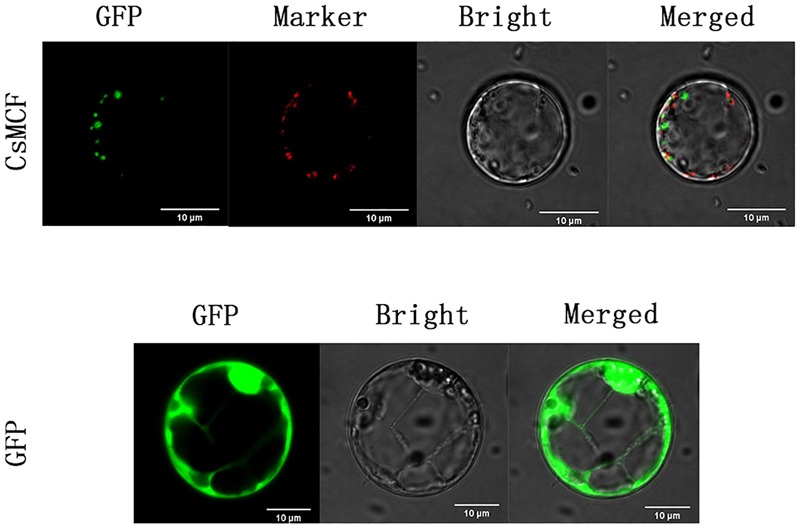
Co-localization of mitochondria of CsMCF-pGII-EGFP protein in rice protoplasts. Images show rice protoplasts expressing CsMCF-pGII-EGFP (upper row) or pGII-EGFP (bottom row). The upper row from left to right is GFP fluorescence, Mitochondrion marker, Bright-field illumination, and an overlay of GFP and Mitochondrion marker are shown. The bottom row from left to right is GFP fluorescence, Bright-field illumination, and an overlay of GFP and Bright-field illumination are shown. Scale bars, 10 μm.

To confirm whether *CsMCF* is located in mitochondria, we co-located the mitochondria (Figure 3). The results showed that *CsMCF* was not located in mitochondria.

### Expression Patterns of *CsMCF* in Response to PM Treatment

**FIGURE 4:**
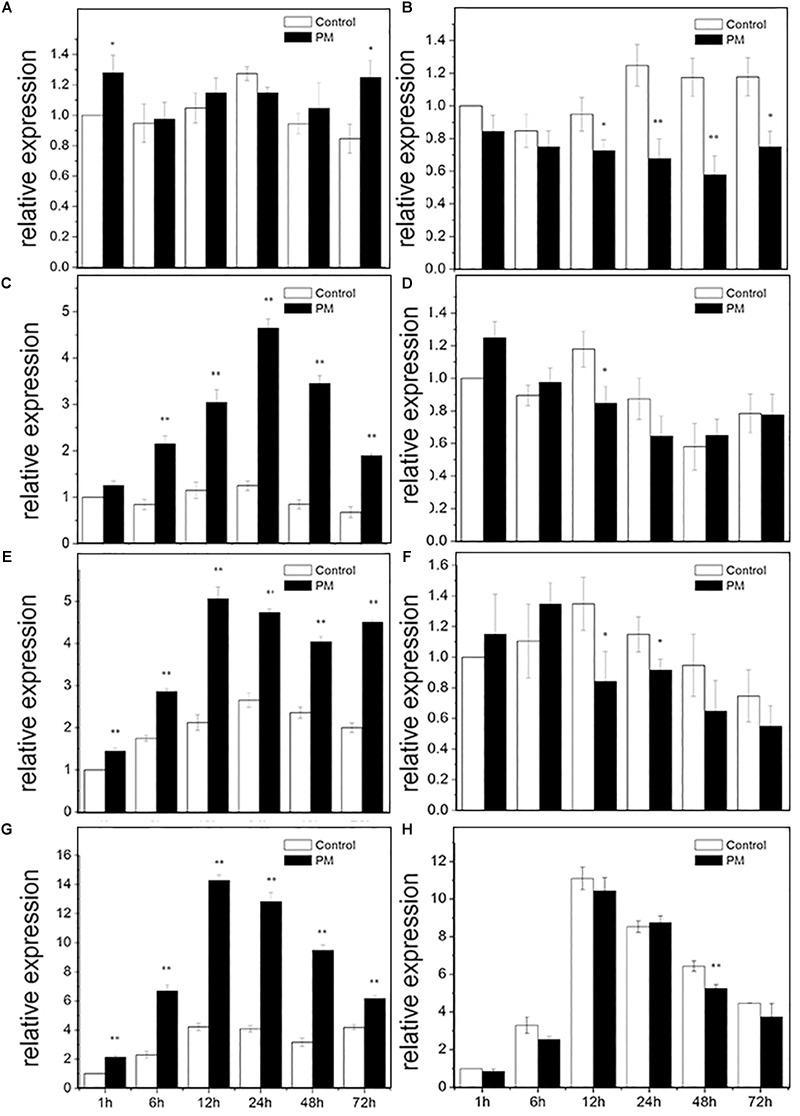
Expression pattern of *CsMCF* in response to propamocarb stress. **(A)** Roots of D0351. **(B)** Roots of D9320. **(C)** Stem of D0351. **(D)** Stem of D9320. **(E)** Leaf of D0351. **(F)** Leaf of D9320. **(G)** Fruit of D0351. **(H)** Fruit of D9320. The “^∗^” presents the value is extremely significant at 0.05 level based on student *t*-test. The “^∗∗^” presents the value is extremely significant at 0.01 level based on student *t*-test.

RT-PCR was used to analyze the expression pattern of *CsMCF* following PM treatment of the low- and high-PM residual cultivars “D0351” and “D9320” (Figure 4). In “D0351” roots, *CsMCF* expression at all time points, except at 24 h, was higher than that of the control, but the difference was not significant. The expression levels showed significant changes only at 1 and 72 h and were 1.28- and 1.48-fold that of the control, respectively (Figure 4A). In “D9320,” the expression levels at each time point were lower than those of the control, and the difference was extremely significant at 24 h and 48 h; the levels were 0.54- and 0.49-fold that of the control (Figure 4B). Gene expression in the stems of “D0351” first increased and then decreased, gradually increasing from 1 h to 24 h and reaching a maximum peak at 24 h of 3.72-fold of that of the control; then, from 24 to 72 h, the expression decreased, and the 72 h expression level of *CsMCF* was 2.81-fold that of the control, lower than the maximum value at 24 h by 0.59-fold (Figure 4C). In the stems of “D9320,” the *CsMCF* expression level was significantly lower than that of the control group at 12 h and was 0.72-fold that of the control group. There was no significant difference in expression at the other time points compared to that of the control group. The expression level ranged from 0.65 to 1.25, showing a linear distribution (Figure 4D). *CsMCF* expression in the leaves of “D0351” was extremely significantly upregulated at all time points compared with that of the control and reached a maximum of 5.07 at 12 h. At this time, the difference between the two groups was the greatest, and expression was 2.39-fold that of the control (Figure 4E). In “D9320” leaves, *CsMCF* was downregulated only at 12 and 24 h, and the difference was the greatest at 12 h, with a value of 0.62-fold of that of the control. There were no significant differences at the other time points. The overall linear distribution was similar to that of the stem (Figure 4F). The expression patterns of *CsMCF* in “D0351” and “D9320” fruits were basically the same, showing a trend of first increasing and then decreasing. The maximum expression of *CsMCF* in “D0351” fruits was 14.28 at 12 h. After 12 h, expression decreased, but the expression level at each time point was still higher than that of the control, and the difference at 12 h between the experimental group and control group was the most significant, with an expression value of 3.39-fold that of the control (Figure 4G). *CsMCF* expression in “D9320” fruits was only slightly higher than that of the control at 24 h (1.03-fold higher than that of the control group). The expression at other time points was lower than that of the control group, and the difference was extremely significant at 48 h, with expression of 0.82-fold that of the control (Figure 4H). *CsMCF* levels in the stem and leaf of “D0351” were higher than those in the same parts of “D9320.” *CsMCF* expression levels in stems and leaves of “D0351” were significantly higher than those of “D9320” at the same time points, especially at 24 h in the stems and 12 h in the leaves. The expression of *CsMCF* in “D0351” was 7.19- and 6.02-fold that in “D9320.” *CsMCF* was specifically expressed in the stems and leaves of “D0351” compared with “D9320.”

### Analysis of *CsMCF* Expression Under Different External Factors

**FIGURE 5:**
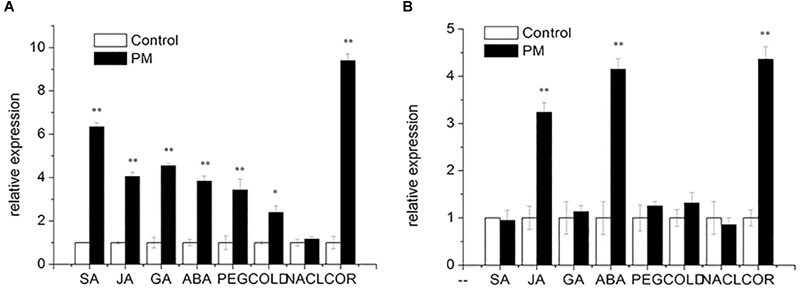
Expression pattern of *CsMCF* under different stress of “D0351” and “D9320.” **(A)** Cucumber variety “D0351.” **(B)** Cucumber variety “D9320.” SA (salicylic acid). JA (jasmonic acid). GA (gibberellic acid). ABA (abscisic acid). PEG (drought stress). COLD (low temperature stress). NACL (salt stress). COR (disease treatment). The “^∗^” presents the value is extremely significant at 0.05 level based on student *t*-test. The “^∗∗^” presents the value is extremely significant at 0.01 level based on student *t*-test.

Salicylic acid, JA, GA and ABA were added at the three true-leaf stages of “D0351” and “D9320.” Then, the expression patterns of *CsMCF* were analyzed. The results showed that (Figure 5) following SA treatment, *CsMCF* expression was extremely significantly upregulated in the low-PM-residue cultivar “D0351,” and the expression was 6.35-fold that of the control. In the high-PM-residue cultivar “D9320,” *CsMCF* expression was not significantly different from that of the control. Under JA induction, the expression patterns of *CsMCF* in “D0351” and “D9320“ were the same, showing an extremely significant upregulation, but *CsMCF* expression in “D0351” was higher than that in “D9320” and showed a 4.05-fold increase in “D0351” and a 3.24-fold increase in “D9320.” After GA treatment, *CsMCF* showed different expression patterns in “D0351” and “D9320.” The *CsMCF* level in “D0351” was extremely significantly upregulated and was 4.55-fold that of the control. The *CsMCF* level in “D9320” was only 1.13-fold that of the control, revealing no significant difference. Following ABA treatment, *CsMCF* expression in “D0351” and “D9320” was extremely significantly upregulated, and the *CsMCF* level in “D9320” was higher than that in “D0351” and was 4.15- and 3.84-fold that of the control, respectively. Thus, after SA and GA induction, the expression pattern of *CsMCF* was significantly different between “D0351“ and “D9320,” which may be one of the reasons for the difference in resistance between “D0351” and “D9320.”

To study the function of *CsMCF* in stress resistance, we applied abiotic stresses, such as PEG, low temperature and multiple salt treatments, to the three true-leaf stages of “D0351” and “D9320.” The expression patterns of *CsMCF* were analyzed, as shown in Figure 5. Under PEG stress, *CsMCF* expression in “D0351” was extremely significantly upregulated and was 3.43-fold that of the control, but there was no significant difference between *CsMCF* expression in “D9320” and the control. At a low temperature, *CsMCF* expression in “D0351” was extremely significantly upregulated and was 2.40-fold that of the control. The expression of *CsMCF* in “D9320” was not significantly different from that of the control. Under multiple salt stresses, there was no significant difference between *CsMCF* and the control in “D0351” and “D9320.” The results showed that under different types of stress, the expression levels of *CsMCF* in “D0351” and “D9320” were different, which might be related to the different abilities of “D0351” and “D9320” to resist PM.

Diseases are biological stressors that have an important impact on plants. As shown in Figure 5, *CsMCF* responded positively to the stress of *C. cassiicola Wei* (Cor), and *CsMCF* expression in “D0351” was extremely significantly increased to 9.40-fold that of the control group. In “D9320,” *CsMCF* expression was extremely significantly upregulated, but the upregulation range was lower than that in “D0351,” in which the expression was only 4.36-fold that of the control. Therefore, *CsMCF* has a unique expression pattern in the low-residual cultivar “D0351” under Cor stress and can respond positively to Cor.

### Construction of the *CsMCF* Expression Vector and Genetic Transformation of Cucumber

**FIGURE 6:**
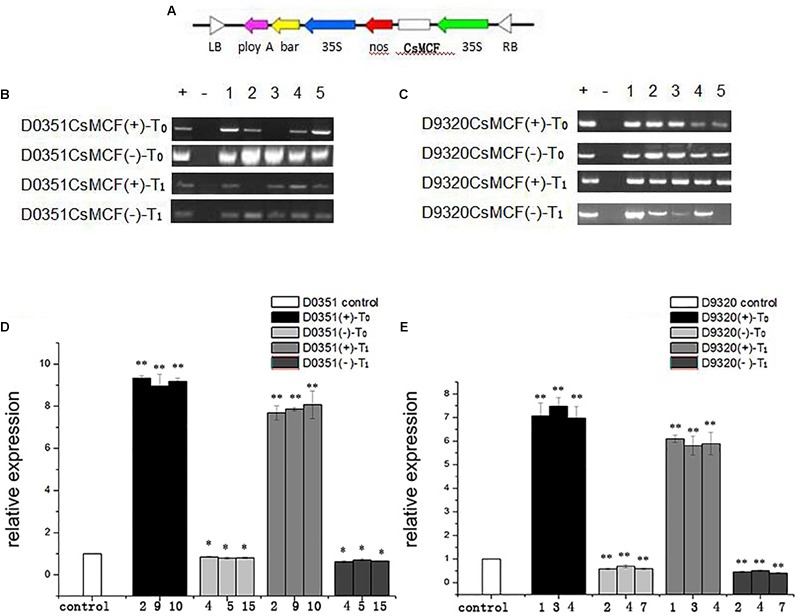
Molecular biological verification of transgenic plants. **(A)** Construction of plant vector pCXSN-CsMCF. **(B)** “D0351” Transgenic plants T_0_ and T_1_ were identified by PCR. **(C)** “D9320” Transgenic plants T_0_ and T_1_ were identified by PCR. **(D)** Relative transcript levels of *CsMCF* in “D0351” transgenic plants T_0_ and T_1_. The three OE lines with the highest expression of *CsMCF* (lines 2, 9, and 10), the AS lines with the lowest expression (lines 4, 5, and 15). **(E)** Relative transcript levels of *CsMCF* in “D9320” transgenic plants T_0_ and T_1_. The three OE lines with the highest expression of *CsMCF* (lines 1, 3, and 4), the AS lines with the lowest expression (lines 2, 4, and 7). The “*” presents the value is extremely significant at 0.05 level based on student *t*-test. The “**” presents the value is extremely significant at 0.01 level based on student *t*-test.

**FIGURE 7:**
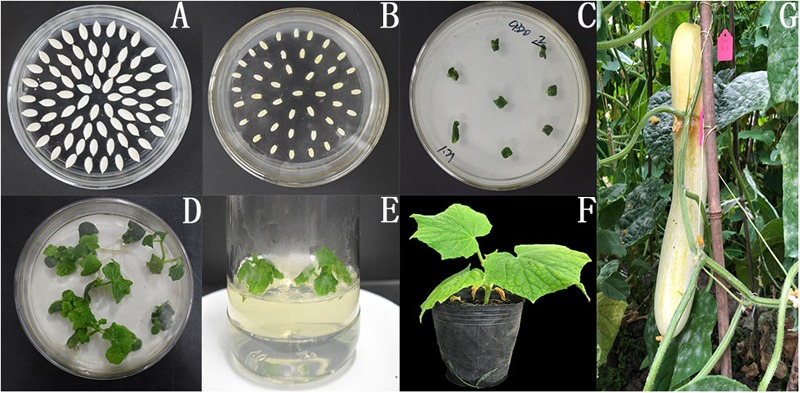
pCXSN-CsMCF genetic transformation of cucumber. **(A)** Cucumber seed. **(B)** Co-culture. **(C)** Screening culture. **(D)** Plant regeneration. **(E)** Rooting culture of resistant seedlings. **(F)** Regeneration of resistant seedlings. **(G)** Seed of transgenic plants.

We generated the overexpression vectors *CsMCF* (+)-PCXSN and *CsMCF* (-)-PCXSN under the control of the strong constitutive CaMV35S promoter (Figure 6A). The overexpression vectors *CsMCF* (+)-PCXSN and *CsMCF* (-)-PCXSN were successfully transferred into “D0351” and “D9320” using cucumber genetic transformation technology (Figure 7).

DNA from the leaves of transgenic plants was extracted as a template, and primers were designed based on the pCXSN vector for PCR amplification. The pCXSN-CsMCF (+) plasmid was used as a positive control, and water was used as a negative control. The results shown in Figure 6B,C indicate the target fragments were approximately 1200 bp in the positive control and some resistant plants, while no target bands were found in the negative control, indicating that the pCXSN-CsMCF plasmid had been integrated into the cucumber genome. T_0_ eliminate false positives in resistant plants and to ensure the integrity and accuracy of the experiment, we extracted RNA from the leaves of transgenic positive plants and non-transgenic plants and performed reverse transcription to convert this template into cDNA for qRT-PCR identification. The results showed that in “D0351,” *CsMCF* expression was significantly increased after transfection of *CsMCF* (+) (OE2, OE9 and OE10). The expression of *CsMCF* in the T_0_ and T_1_ generations was approximately 9.16- and 7.87-fold that in the wild type, respectively. After transfection of *CsMCF* (-) (AS4, AS5, and AS15), the expression of *CsMCF* was downregulated and was approximately 0.82- and 0.67-fold that of the wild type (Figure 6D). In “D9320,” *CsMCF* expression was upregulated after *CsMCF* (+) transfer (OE1, OE3, and OE4), and the expression of the T_0_ and T_1_ generations was approximately 7.18- and 5.93-fold that of the wild type. After *CsMCF* (-) transfer (AS2, AS4 and AS7), the expression of T_0_ and T_1_ was approximately 0.62- and 0.46-fold that of the wild type (Figure 6E). The introduction of exogenous genes affected the normal expression of *CsMCF* in cucumber, and the expression level was changed. The difference in expression patterns was extremely significant, which indicated that *CsMCF* (+) and *CsMCF* (-) had been successfully transferred into cucumber. The three overexpression strains with the highest expression and the three antisense strains with the lowest expression were screened for the detection of the PM residues and the determination of physiological and biochemical indicators.

### PM Residue Analysis of *CsMCF*-Overexpressing Plants

**FIGURE 8:**
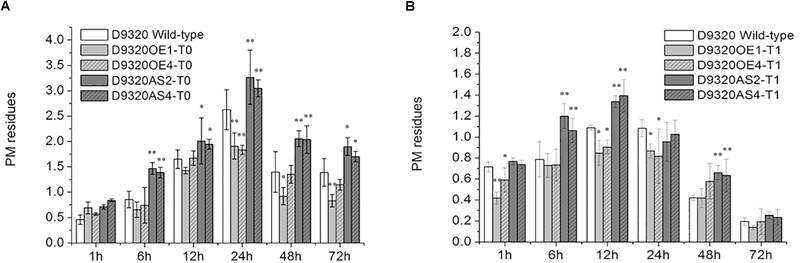
Detection of PM residues in transgenic Plants. **(A)** Quantitative analysis of residues in T_0_ transgenic plants using gas chromatography. **(B)** Quantitative analysis of residues in T_1_ transgenic plants using gas chromatography. The “^∗^” presents the value is extremely significant at 0.05 level based on student *t*-test. The “^∗∗^” presents the value is extremely significant at 0.01 level based on student *t*-test.

Plants with similar expression levels were screened from the “D9320” plants identified by PCR and qRT-PCR. The PM residues in fruits were determined after treatment with PM (Figure 8 and Supplementary Figures S3, S4). The results showed that in *CsMCF* (+)-overexpression T_0_ plants (OE1 and OE4), the PM residues in cucumber fruits at five time points (6–72 h), but not the 1 h time point, were lower than those in the wild-type control. The PM residues of OE1 lines increased rapidly at 6 h. The maximum value was 1.91 mg/kg at 24 h, and at 24 h and 72 h, the PM residues of OE1 lines fruits were extremely significantly lower than those of the wild type and were 0.73- and 0.60-fold that of the wild type, respectively. The average residue of OE1 lines fruits at 6 time points was 1.07 mg/kg, which was 0.32 mg/kg lower than the average wild-type residue. The PM residues of OE4 lines reached a maximum value of 1.84 mg/kg at 24 h and at 24 h, the PM residues of OE4 lines were extremely significantly lower than those of the wild type and were 0.69-fold that of the wild type. We concluded that *CsMCF* (+) transfer could effectively reduce the PM residues in cucumber fruits. At six time points during 1–72 h, the fruit residues of *CsMCF* (-)-overexpression plants (AS2 and AS4) were significantly higher than those of wild-type plants. The average residue of AS2 lines was 1.90 mg/kg, which was 0.50 mg/kg higher than that of wild-type plants. The average residue of AS4 lines was 1.83 mg/kg, which was 0.43 mg/kg higher than that of wild-type. The overall trend of AS2 lines and AS4 lines is similar. Transfer of the antisense gene increased the PM residues in fruits, which indicated that the antisense gene had an inverse regulatory effect. The AS2 lines PM residues at 6, 24 and 48 h were extremely significantly higher than those of the wild type by 1.71-, 1.24-, and 1.47-fold, respectively, and at the same time, AS4 lines PM residues were 1.62-, 1.16-, and 1.46-fold that of the wild type, respectively.

The variation trend of PM residues in T_1_ plants was the same as that in T_0_ plants, showing a trend of first increasing and then decreasing. However, the PM residues in *CsMCF* (+)-overexpression (OE1 and OE4) and *CsMCF* (-)-overexpression (AS2 and AS4) T_1_ plants decreased at all time points compared with those of T_0_ plants. In the OE1 lines from the T_1_ generation, the PM residues was lower than that of the wild-type plants at 6 time points, reaching a maximum value of 0.87 mg/kg at 24 h and then decreasing significantly. The difference between wild-type and OE1 lines was extremely significant at 1 h; the level of the OE1 lines was 0.59-fold that of the wild-type plants. The residues at 12 h and 24 h were significantly different from those of the wild-type plants, 0.78- and 0.80-fold that of the wild type, respectively. Fruits from the OE1 lines from the T_1_ generation had an average PM residue of 0.57 mg/kg, which was lower than that of the wild type (0.15 mg/kg). Among the six time points of OE4 lines, PM residues were lower than that of the wild type at other times except 48 h. The OE4 lines from the T_1_ generation had an average PM residue of 0.64 mg/kg. The PM residue of *CsMCF* (-)-overexpression plants (AS2 and AS4) at six time points were higher than those of wild type except 24 h. The PM residue of AS2 lines reached a maximum value of 1.34 mg/kg at 12 h and then decreased gradually. The inverse regulatory effect was most significant at 6, 12, and 48 h. The residues of AS2 lines were 1.52-, 1.23-, and 1.57-fold those of the wild type, respectively. The average residue of AS2 lines at six time points was 0.86 mg/kg, which was higher than that of the wild type (0.15 mg/kg). The PM residue of AS4 lines reached a maximum value of 1.39 mg/kg at 12 h and then decreased gradually. The inverse regulatory effect was most significant at 6, 12, and 48 h, the PM residues of AS4 lines were 1.34-, 1.28-, and 1.50-fold those of the wild type, respectively. The average residue of AS4 lines at 6 time points was 0.85 mg/kg, which was 0.70 mg/kg higher than that of the wild type.

### Physiological and Biochemical Indexes Under PM Treatment

#### POD Analysis of *CsMCF*-Overexpression Plants

**FIGURE 9:**
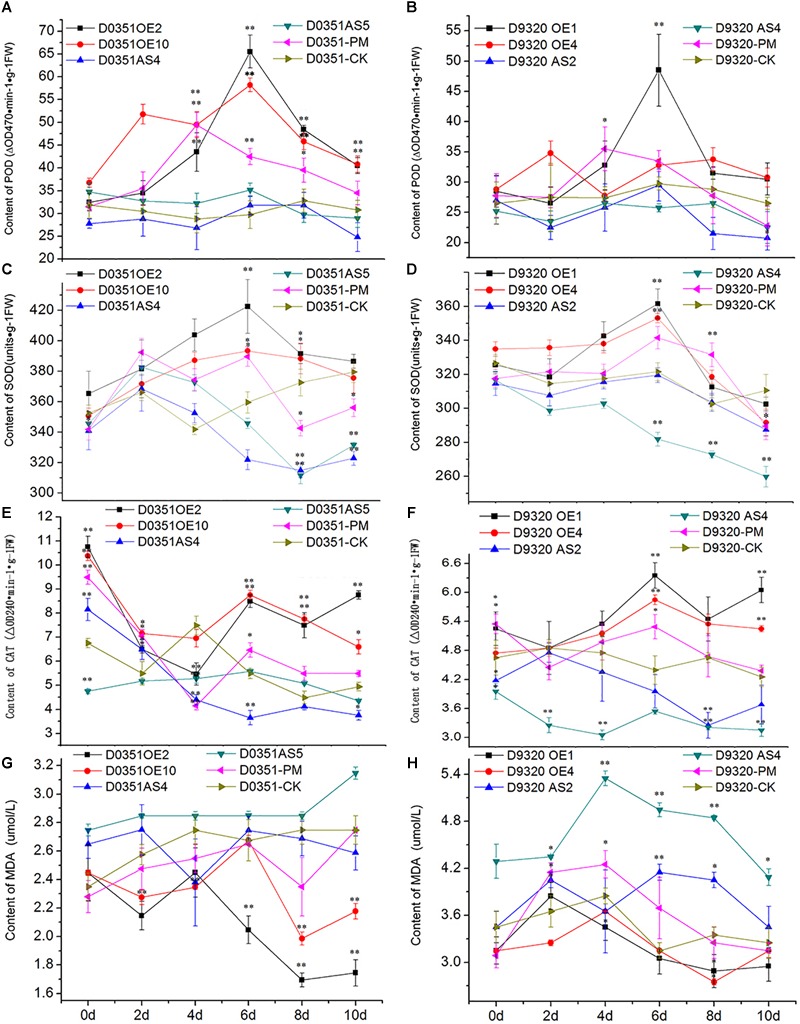
Analysis of physiological indexes under T_0_ stress of PM. **(A)** “0351” POD content. **(B)** “9320” POD content. **(C)** “0351” SOD content. **(D)** “9320” SOD content. **(E)** “0351” CAT content. **(F)** “9320” CAT content. **(G)** “0351” MDA content. **(H)** “9320” MDA content. The “^∗^” presents the value is extremely significant at 0.05 level based on student *t*-test. The “^∗∗^” presents the value is extremely significant at 0.01 level based on student *t*-test.

*CsMCF* is defined as the carrier protein of a peroxidant coenzyme I in cucumber and is closely related to POD activity. As shown in Figure 9A, POD activity in “D0351” OE2 lines increased significantly with time, reaching a maximum value of 65.51 ΔOD470 min^-1^g^-1^ FW on the 6th day and then decreasing. Except for that on the 2nd and 4th days, the POD activity of OE2 lines at other time points was significantly higher than that of wild-type PM-treated plants and higher than that of wild-type distilled water control-treated plants at all time points. The POD activity of OE10 lines reached the maximum value of 58.19 ΔOD470 min^-1^g^-1^FW on the 6th day. The POD activity of OE10 lines was higher than that of wild-type PM-treated plants and wild-type distilled water control-treated plants at all time points. The POD activity of AS4 lines reached the maximum value of 31.75 ΔOD470 min^-1^g^-1^FW on the 6th day, which was lower than that of OE2 lines (33.76 ΔOD470 min^-1^g^-1^FW) and decreased rapidly on the 8th day after PM treatment; the values were significantly lower than those of the wild-type PM treatment group at all time points. The POD activity on the 6th day of stress was slightly higher than that of the wild-type distilled water control, and at other time points, it was lower than that of the control. The POD activity of AS5 lines reached the maximum value of 35.17 ΔOD470 min^-1^g^-1^FW on the 6th day, the POD activity at 5 time points of 2–10 days were lower than wild-type PM treatment group. As shown in Figure 9B, the POD activity in OE1 lines decreased rapidly after reaching the maximum value of 48.49 ΔOD470 min^-1^g^-1^FW on the 6th day but showed little change after 8 days of PM stress, and the POD activity tended to be stable. Except for the POD activity on the 2nd day, which was basically similar to that of the wild-type PM treatment group and wild-type distilled water control group, and the 4th day activity, which was slightly lower than that of the wild-type PM treatment group, the POD activity at all other time points was higher than that of the wild-type PM treatment and wild-type distilled water groups. The pod value of OE4 was lower than that of wild-type PM treatment on the 4th and 6th day, but higher than that of wild-type distilled water groups at other time points. The POD activity of AS2 lines also reached a maximum value of 29.49 ΔOD470 min^-1^g^-1^FW at 6 h, at which time the POD content of was 19.00 ΔOD470 min^-1^g^-1^FW lower than that of OE1 lines and was lower than that of the wild-type PM treatment and wild-type distilled water control groups at all time points. The POD activity of AS4 lines reached a maximum value of 26.49 ΔOD470 min^-1^g^-1^FW at 8 h, the POD activity at all time points was lower than that of the wild-type PM treatment and wild-type distilled water groups. By comparing Figure 9A,B, we observed that *CsMCF* could affect the activity of the POD enzyme. Transgenic genes enhanced the POD activity, and transgenic antisense vectors weakened the POD activity.

#### SOD Analysis of *CsMCF*-Overexpression Plants

SOD can effectively remove harmful substances produced by cucumber metabolism and enhance plant immunity. As shown in Figure 9C, the SOD content of OE2 lines first increased and then decreased. Although the SOD content on the 2nd day was lower than that of the wild-type PM treatment group, SOD levels at the other time points were higher than those of the wild-type PM treatment group, and the SOD content at each time point was significantly higher than that of the wild-type distilled water control. The SOD content of OE10 reached the maximum value of 393.29 U⋅g^-1^ on the 6th day, and the SOD content at most of the time points was higher than that of the wild-type PM treatment group and wild-type distilled water control. The SOD content of AS4 lines was lower than that of OE2 lines, OE10 lines and “D0351” wild-type PM-treated plants at all time points. The SOD content of the wild-type distilled water control group was basically similar, except for that on the 2nd day, and the content on the 4th day was slightly higher than that of the wild-type distilled water control group. The values at other time points were significantly lower than those of the wild-type distilled water control. The SOD content of AS5 lines extremely significantly lower than OE2 lines, OE10 lines, “D0351” wild-type PM-treated plants and wild-type distilled water control on the 8th and 10th days. As shown in Figure 9D, the SOD content of OE1 lines reached the maximum value of 361.33 U⋅g^-1^ on the 6th day. Except for those on the 2nd and 8th days, the SOD contents at the other time points were higher than those of the wild-type PM treatment group. The SOD content was slightly lower than that of the wild-type distilled water control only on the 10th day. The SOD content of OE4 reached the maximum value of 352.99 U⋅g^-1^ on the 6th day. The SOD content of AS2 reached the maximum value of 319.07 U⋅g^-1^ on the 6th day, but it was much lower than that of “D9320” *CsMCF* (+)-overexpression plants, and the SOD contents at all time points were lower than those of the wild-type PM treatment group. On the 4th, 6th, and 8th days, the SOD contents were basically similar to those of the wild-type distilled water control group, while the SOD contents at the other time points were significantly lower than those of the control. The SOD content of AS4 was lower than wild-type PM treatment group and wild-type distilled water control group from the 2nd to 10th days, and reached a extremely significantly level from the 6th day to the 10th day.

The transfer of *CsMCF* changed the plant SOD activity. In plants with high expression of *CsMCF*, the SOD content was high, while in plants with low expression of *CsMCF*, the SOD content was low. The SOD content of “D0351” *CsMCF* (+)-overexpression plants was significantly higher than that of “D9320.” The SOD contents in the two genotypes of cucumber showed significant differences. We hypothesized that SOD directly participated in the metabolic response to the cucumber against PM. The difference in SOD content may be one of the reasons for the difference in PM residues on the fruits of the two varieties.

#### CAT Analysis of *CsMCF*-Overexpression Plants

CAT can decompose H_2_O_2_ into H_2_O and O_2_ under adverse conditions and thus scavenges H_2_O_2_ to maintain the stability of the membrane. As shown in Figure 9E, the CAT content in OE2 lines was significantly higher than that in wild-type PM-treated plants at all time points, except on the 2nd day. The CAT content at all other time points, except for the 4th day, were higher than those in the wild-type distilled water control. The CAT content of OE10 was higher than that in wild-type PM-treated plants at all time points, and except for the 4th day, the CAT content was higher than wild-type distilled water control at the other time points. The CAT content in AS4 lines was basically similar to that in the wild-type PM-treated plants on the 4th day and was significantly lower than that in the wild-type PM-treated plants at the other time points. The CAT content of AS5 lines reached the maximum value of 5.58 ΔOD240 min^-1^g^-1^FW on the 6th day. The overall trend of change is stable. The CAT content of “D0351” *CsMCF* (+)-overexpression plants was higher than that of “D0351” *CsMCF* (-)-overexpression plants at all time points, indicating that the CAT content was affected by the regulation of *CsMCF*. As shown in Figure 9F, the CAT contents of OE1 lines and OE4 lines were higher than those of “D9320” wild-type PM-treated plants and wild-type distilled water control plants from 2nd to 10th days. OE1 lines reached the maximum value of 6.35 ΔOD240 min^-1^g^-1^FW on the 6th day. OE4 lines reached the maximum value of 5.85 ΔOD240 min^-1^g^-1^FW on the 6th day. The CAT content of AS2 lines was significantly lower than that of other treatments, although it was similar to that of the wild-type PM treatment and wild-type distilled water control plants on the 2nd day. The CAT content of AS4 lines was significantly lower than that of other treatments at all time points. The overall trend of the CAT content in “D0351” *CsMCF* (+)-overexpression plants and “D9320” *CsMCF* (+)-overexpression plants was similar, but the CAT content in “D0351” *CsMCF* (+)-overexpression plants was higher than that in “D9320” *CsMCF* (+)-overexpression plants at each time point. These findings indicate that the CAT level might be related to the detoxification of PM in cucumber fruits.

#### MDA Analysis of *CsMCF*-Overexpression Plants

MDA analysis can reveal the peroxidation degree of the plant cell membrane as the MDA content indicates the degree of damage to the plant cell membrane. The higher the MDA content is, the more damage to the plants. As shown in Figure 9G, starting from the 4th day of PM exposure, the MDA content of “D0351” *CsMCF* (+)-overexpression (OE2 and OE10)plants was significantly lower than that of “DOk sir” wild-type PM-treated plants and wild-type distilled water control plants. The MDA content of AS4 lines was slightly lower than that of “D0351” wild-type PM-treated plants on the 4th and 10th days and was higher than that of wild-type PM-treated plants at other time points, and the MDA content was significantly higher than that of wild-type distilled water control plants within 6 days of PM treatment. The MDA content of AS5 lines was significantly higher than those of wild-type PM-treated plants and wild-type distilled water control plants at all time points. As shown in Figure 9H, the MDA content of OE1 lines was similar to that of “D9320” wild-type PM-treated plants at the beginning of the experiment and then decreased at all time points. The MDA content at the other time points, except for the 2nd day, was lower than that of “D9320” wild-type distilled water control. The MDA content of OE4 lines was lower than that of wild-type PM-treated plants from 2nd to 10th days, and was lower than that of wild-type distilled water control plants at all time points. The MDA content of “D9320” *CsMCF* (-)-overexpression (AS2 lines and AS4 lines) plants was significantly higher than that of wild-type PM treatment and wild-type distilled water control plants except for the AS2 lines on the 4th day. The MDA content of “D9320” *CsMCF* (-)-overexpression plants was higher than that of “D9320” *CsMCF* (+)-overexpression plants at all time points. As shown in Figure 9G,H, the MDA content in the four treatments of “D0351” was lower than that in “D9320,” which may be due to the differences in the MDA contents of the different varieties. However, in “D0351” and “D9320,” the transfer into *CsMCF* (+) reduced the MDA content. This finding indicated that *CsMCF* (+) could reduce the degree of membrane peroxidation and improve the resistance of cucumber to PM.

## Discussion

In this study, a differentially upregulated gene, *CsMCF*, was identified from previous transcriptome sequencing results of PM exposure experiments. The subcellular localization of this protein is in the cytoplasm, where various organelles and many types of proteins reside. Usually, there are some enzymes with redox and detoxification effects and proteins related to plant stress resistance. We hypothesized that *CsMCF* may have redox or detoxification effects and can thus respond to biotic and abiotic stresses and improve cucumber resistance to stress. PlantCARE was used to analyze the promoter region 2000 bp upstream of the *CsMCF* ATG. Multiple MYB binding sites and recognition sites were found in the promoter region. MYB transcription factors participate in plant secondary metabolism, hormones and environmental responses, which are closely related to plant resistance to adverse environments (Uimari, 1997; Chen et al., 2003; Lea et al., 2007) *CsMCF* effectively, reduced the PM residues in cucumber fruits. When cucumber is exposed to PM, stress signals may stimulate the expression of MYB transcription factors. MYB-related genes play a role in regulating the plant’s internal environment in response to environmental changes, triggering a series of physiological and biochemical changes, and improving the resistance of cucumber to PM.

The expression patterns of *CsMCF* in cucumber genotypes with different PM residual levels were significantly different. After PM treatment, *CsMCF* expression levels in the roots, stems, leaves and fruits of “D0351” were higher than those of *CsMCF* in the same organs of “D9320” at all time points. The order of expression change in each organ was as follows: fruit > leaf > stem > root. The stems and leaves were sprayed with PM, and the results showed that these organs are involved in PM transport, as reflected by the finding that the most obvious differences in expression were found in these organs. The results were consistent with those of transcriptome sequencing (Wu et al., 2013). These findings suggested that the expression of *CsMCF* was specifically upregulated in the low-PM-residue cucumber cultivar “D0351,” and actively responded to the PM treatment. We considered that the effect of the *CsMCF* gene might be one important reason for the difference in PM residues between the two genotypes of cucumber. Previous research showed that *CsABC19* was mainly expressed in fruits. The tissue specificity analysis in this study also showed that the relative expression of *CsMCF* was the highest in fruits. Previous research analyzed the expression pattern of *CsABC19* in “D0351” and “D9320” exposed to PM. The results showed that *CsABC19* was mainly expressed in “D0351” (Meng et al., 2016), and the expression pattern of *CsMCF* in this study was similar to that of *CSABC19*. Based on the above results, *CsMCF* is constitutively expressed and shows varietal differences and organizational differences. This gene can respond positively to PM treatment and shows tolerance to PM exposure. Thus, we concluded that *CsMCF* participates in the process of detoxification or degradation of PM and plays an important role in reducing the PM residue in fruits.

Because the fruit is the edible part of cucumber and *CsMCF* expression is the highest in the fruit, fruits of T_0_ and T_1_ generation plants with the same growth potential and similar expression were selected to detect the PM residues. The results showed that the metabolic pattern of PM in the T_0_ and T_1_ generations was the same, first increasing and then decreasing. From the above analysis of *CsMCF* gene expression (Figure 4), we found that after PM treatment, *CsMCF* expression in “D9320” increased rapidly at 12 h. After 12 h, the residues of *CsMCF* (+)-overexpressing cucumber fruits were significantly or extremely significantly lower than those of *CsMCF* (-)-overexpressing plants and wild-type plants. These findings indicated that the residue level was negatively correlated with the gene expression level. Previous research showed the rate of change in PM residues in fruits. The rate of change in the PM residues in cucumber fruits was the highest at 24 h (Zhao, 2013). In this study, except for *CsMCF* (-)-overexpression plants in the T_1_ generation, the *CsMCF* (+)-overexpression plants in the T_0_ and T_1_ generations and *CsMCF* (-)-overexpression plants in the T_0_ generation reached the maximum rate at 24 h, followed by a decrease, and the *CsMCF* (-)-overexpression plants in the T_1_ generation reached the maximum at 12 h. These results indicated that *CsMCF* could respond to PM exposure. Transfer of *CsMCF* (+) could effectively reduce the PM residue, which may be related to the detoxification and metabolism of PM in cucumber. The PM residues were higher than those of wild-type plants at all time points after transfer of *CsMCF* (-). The introduction of antisense genes may have inhibited enzymes or genes related to degradation and metabolism in fruits and affected the detoxification metabolism of PM in fruits. *CsMCF* can effectively reduce the PM residue in “D9320” plants at the T_1_ generation, which indicated that the gene had certain genetic stability. These results are consistent with the previous study (Liu et al., 2018).

Leaves showed the greatest differential expression of *CsMCF* in “D0351” and “D9320,” and thus, the physiological and biochemical indexes of leaves were selected for analyses. Because the physiological and biochemical indexes of the leaves changed little within 0–72 h, differences could not be distinguished, and therefore, the detection time to determine the physiological and biochemical indexes was extended. We selected days 0, 2, 4, 6, 8, and 10 as the sampling times based on preparatory experiments. When cucumber is subjected to external stress, the levels of membrane system protective enzymes (mainly SOD, POD, and CAT) *in vivo* will increase. SOD can catalyze the disproportionation of O_2_ to H_2_O_2_, and then POD and CAT can reduce it to H_2_O, which has an important role in scavenging oxygen free radicals, thereby increasing the metabolic capacity of plants to toxic substances (Gémes et al., 2011). In this experiment, the contents of SOD, POD and CAT in cucumber were increased by the transfer of *CsMCF* (+) into cucumber, while the contents of SOD, POD and CAT following transfer of *CsMCF* (-) were significantly lower than those in the corresponding wild-type plants. These findings indicated that *CsMCF* affected the SOD, POD and CAT contents in cucumber. This treatment could regulate SOD, POD and CAT contents by increasing the expression of *CsMCF* and inducing the accumulation of H_2_O_2_ in cucumber fruits. Reactions reducing H_2_O_2_ to H_2_O could improve cucumber metabolism of PM and effectively reduce PM residues. The results were consistent with those of previous studies (Davies and Swanson, 2001; Chaitanya et al., 2002; Mazorra et al., 2002) and other studies on POD activity after abiotic stress. MDA is one of the most important products of membrane lipid peroxidation. The MDA content is a commonly used index in the study of plant senescence physiology and resistance physiology. The degree of membrane lipid peroxidation indicated by MDA can indirectly measure plant stress resistance (Dionisio-Sese and Tobita, 1998). Our results showed that *CsMCF* could effectively regulate the MDA content in cucumber fruits. Transferring *CsMCF* (+) significantly reduced the MDA content in cucumber fruits, but the opposite results were obtained after the transfer of *CsMCF* (-). The MDA content could be reduced by transferring *CsMCF* (+) into cucumber to reduce the degree of membrane peroxidation of cucumber, thereby improving the antioxidant capacity of cucumber and its resistance to PM.

Hormones play an important role in the adaptation and signal transduction of plant stress responses. SA can induce an increase in membrane system protective enzymes (mainly SOD, POD, and CAT) in leaves, which can scavenge oxygen free radicals and enhance the antioxidant capacity of plants (Surassawade et al., 2012). Under stress conditions, a GA synthesis system can be activated and synthesize high levels of GAs, thereby enhancing the ability of plants to resist stress (Finkelstein et al., 2002). JA is an endogenous growth regulator in higher plants. During stress resistance, signal molecules induce the expression of resistance genes, which is closely related to plant resistance (Hao et al., 2012). ABA is known as a stress hormone. External stimulation can increase ABA rapidly and enhance stress resistance in plants. The results of this study revealed that after JA and ABA treatment, the expression patterns of *CsMCF* in “D0351” and “D9320” were similar, but the expression of *CsMCF* in “D0351” was higher than that in “D9320,” which indicated that “D0351” was more sensitive to hormone induction. After SA and GA induction, the expression pattern of *CsMCF* was significantly different between “D0351” and “D9320.” SA can alleviate acute renal injury induced by Paraquat (PQ), and its mechanism may be related to activation of Nrf2-ARE antioxidant signaling pathways (Wang et al., 2017). The detoxification and metabolism of gibberellin can alleviate the harm of herbicides to rice (Wang, 2018). This finding may be related to the low residual PM content in “D0351,” but further experiments are needed to confirm that the tolerance of cucumber to PM can be improved by regulating the hormone pathway during PM metabolism. The process may also involve defense and stress-induced response regulatory elements. The results showed that *CsMCF* expression was significantly different between “D0351” and “D9320” after PEG treatment and low temperature treatment. The expression patterns of *CsMCF* in “D0351” and “D9320” under salt stress were similar. The adaptation and domestication of cucumber under adverse conditions can increase the resistance and tolerance of cucumber and ultimately improve the ability of the cucumber body to resist threats. These processes may also play an important role in plant resistance to PM exposure. The expression of *CsMCF* in “D0351” and “D9320” was significantly upregulated under Cor stress, and *CsMCF* expression in “D0351” was higher than that in “D9320,” which indicated that *CsMCF* could respond positively to biological stresses such as Cor. “D9320” has been proven to be a highly resistant cultivar (Liu, 2017). *CsMCF* may be involved in resistance to *Corynespora* leaf spot, but whether this gene is a disease resistance gene and the specific regulatory mechanism underlying cucumber disease resistance requires further analyses.

**FIGURE 10:**
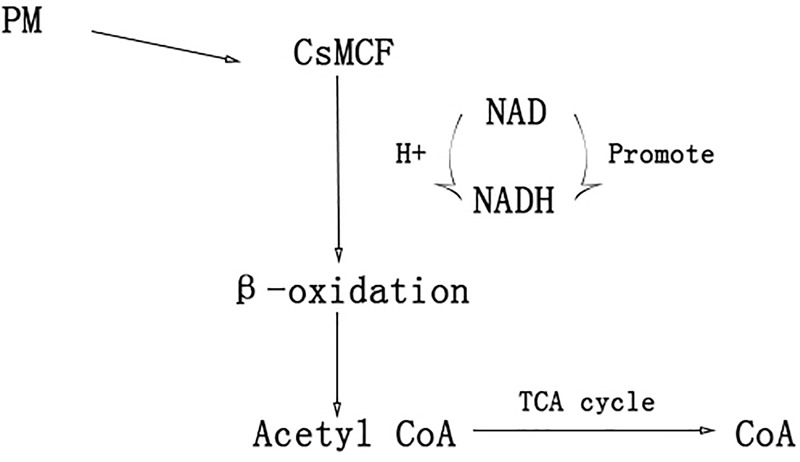
A hypothetical model of *CsMCF* for reducing the residue of PM.

In humans, plants and fungi, members of the mitochondrial vector family can transport NAD into mitochondria, plastids and peroxisomes (Todisco et al., 2006; Palmieri et al., 2008; Agrimi et al., 2012a,b; Bernhardt et al., 2012) NAD and its phosphorylated form, as receptors and donors in redox reactions, are related to the formation and removal of harmful reactive oxygen species (Dröge, 2002; Mittler, 2002). Peroxisome NAD carriers provide NAD for the β-oxidation system, which can convert NAD to NADH. The NAD+/NADH ratio directly controls cell rhythms, aging, cancer and death. NADH can increase the level of energy metabolism, repair cell damage, enhance the cell stress response and reduce the toxic damage of drugs to normal tissues. Another transport scheme for peroxisome NAD carriers is CoA output. When acetyl-CoA, the final product of β-oxidation, is further metabolized through the glyoxylic acid cycle, a high level of CoA (Graham, 2008; van Roermund et al., 2016) is released. CoA can activate immunity to some extent. CoA helps the immune system to detoxify harmful substances, activate white blood cells, promote the synthesis of hemoglobin, participate in the synthesis of antibodies, promote the utilization of coenzyme Q10 and coenzyme I, and alleviate the toxic and side effects caused by antibiotics and other drugs (Fulda et al., 2004) *CsMCF* can effectively reduce propoxur residue in cucumber fruits through two mechanisms. The first mechanism is to increase *CsMCF* expression to enhance NAD transport, promote conversion between NAD and NADH, enhance the cell stress response and reduce the toxicity of drugs to normal tissues through NADH. This process reduces the toxic and side effects of PM on cucumber. The second mechanism involves upregulation of *CsMCF*, which enhances the catalytic transport of CoA. CoA activates the immune system of cucumber and enhances the detoxification of PM (Figure 10). The aims of this study were to explore the performance of *CsMCF* under PM stress and help elucidate the mechanism underlying the production of low residual PM contents in cucumber, and the results will contribute to the breeding of new cucumber varieties with a low PM residues and provide a theoretical basis for breeding low-PM residue varieties of other crops.

## Conclusion

*CsMCF* was cloned, and the full-length gene was 1026 bp, encoding 341 amino acids. This gene had the highest homology with *C. moschata* (XM 023140220.1), and its subcellular localization was in the cytoplasm. *CsMCF* expression was different among varieties and showed tissue specificity. This gene was mainly expressed in the low-PM-residue cultivar “D0351,” and the highest expression was found in fruits. The time points for regulation and control are approximately 6–48 h after PM exposure. Transferring *CsMCF* (+) into cucumber effectively reduced the PM residue, increased the activities of protective enzymes (POD, SOD, CAT) in the cucumber inner membrane system, and reduced the MDA content, which is related to the degree of membrane lipid peroxidation. Transferring *CsMCF* (-) into cucumber had the opposite effect. *CsMCF* could also respond to SA, GA, PEG, low temperature and Cor stress. The response of *CsMCF* to SA, GA, PEG and Cor stress in “D0351” was significantly higher than that of “D9320.”

## Data Availability

All datasets for this study are included in the manuscript and the Supplementary Files.

## Author Contributions

FZ and ZQ conceived and designed the study. FZ, MX, and XZ performed the experiments. SY and DL analyzed the sequencing data. FZ wrote the entire manuscript. ZQ edited the manuscript.

## Conflict of Interest Statement

The authors declare that the research was conducted in the absence of any commercial or financial relationships that could be construed as a potential conflict of interest.

## Footnotes

1 http://www.icugi.org/

2 https://www.ncbi.nlm.nih.gov/

3 https://web.expasy.org/cgi-bin/protparam/protparam

4 https://web.expasy.org/protscale/

5 http://www.cbs.dtu.dk/services/SignalP/

6 https://npsa-prabi.ibcp.fr/cgi-bin/npsa_automat.pl?page=/NPSA/npsa_sopma.html

7 http://bioinformatics.psb.ugent.be/webtools/plantcare/html/

## Supplementary Material

FIGURE S1 Cloning of CDS sequences of *CsMCF* and Sequence analysis. **(A)** Gel electrophoresis of PCR products. **(B)** Enzyme electrophoresis image.

FIGURE S2 The Bioinformatics prediction of *CsMCF* coding protein. **(A)** The conserved domain of *CsMCF* coding protein. **(B)** Protein hydrophobicity prediction. **(C)** Signal peptide of *CsMCF* coding protein. **(D)** The transmembrane region of *CsMCF* coding protein. **(E)** Tertiary structure of protein. **(F)** Secondary protein structure.

FIGURE S3 Detection of PM residues in T_0_ D9320OE3 and D9320AS7 lines plants.

FIGURE S4 Detection of PM residues in T_1_ D9320OE3 and D9320AS7 lines plants.

TABLE S1 All sequences in phylogenetic tree.

## References

[B1] Aebi H. (1984). Catalase in vitro. *Methods Enzymol.* 105 121–126. 10.1016/S0076-6879(84)05016-3 6727660

[B2] Agrimi G. Russo A. Scarcia P. Palmieri F. (2012b). The human gene SLC25A17 encodes a peroxisomal transporter of coenzyme A, FAD andNAD+. *Biochem. J.* 443 241–247. 10.1042/BJ20111420 22185573

[B3] Agrimi G. Russo A. Pierri C. L. Palmieri F. (2012a). The peroxisomal NAD+carrier of Arabidopsis thaliana transports coenzyme A and its derivatives. *J. Bioenerg. Biomembr.* 44 333–340. 10.1007/s10863-012-9445-0 22555559

[B4] Bernhardt K. Wilkinson S. Weber A. P. M. Linka N. (2012). A peroxisomal carrier delivers NAD and contributes to optimal fatty acid degradation during storage oil mobilization. *Plant J.* 69 1–13. 10.1111/j.1365-313X.2011.04775.x 21895810

[B5] Bradford M. M. (1976). A rapid and sensitive method for the quantitation of microgram quantities of protein using theprinciple of protein-dye binding. *Anal. Biochem.* 72 248–254. 10.1016/0003-2697(76)90527-3 942051

[B6] Chaitanya K. V. Sundar D. Masilamani S. Reddy A. R. (2002). Variation in heat stress-induced antioxidant enzyme activities among three mulberry cultivars. *Plant Growth Regul.* 2002 175–180. 10.1023/A:1015092628374

[B7] Chen S. Songkumarn P. Liu J. Wang G. L. (2009). A versatile zero background T-vector system for gene cloning and functional genomics. *Plant Physiol.* 150 1111–1121. 10.1104/pp.109.137125 19403729 PMC2705043

[B8] Chen S. C. Peng S. Q. Huang G. X. (2003). Association of decreased expression of a Myb transcription factor with the TPD(tappingpaneldryness) syndrome in *Heveabrasiliensis*. *Plant Mol. Biol.* 51 51–58. 10.1023/a:1020719420867 12602890

[B9] Davies D. G. Swanson H. R. (2001). Activity of stress-related enzymes in the perennial weed leafy spurge. *Environ. Exp. Bot.* 46 95–108. 10.1016/S0098-8472(01)00081-8

[B10] Dionisio-Sese M. L. Tobita S. (1998). Antioxidant responses of rice seedlings to salinity stress. *Plant Sci.* 135 1–9. 10.1016/s0168-9452(98)00025-9

[B11] Dröge W. (2002). Free radicals in the physiological control of cell function. *Physiol. Rev.* 82 47–95. 10.1152/physrev.00018.2001 11773609

[B12] Finkelstein R. R. Gampala S. S. Rock C. D. (2002). Abscisic acid signaling in seeds and seedlings. *Plant Cell* 14 s15–s45. 10.1105/tpc.010441 12045268 PMC151246

[B13] Foyer C. H. Halliwell B. (1976). The presence of glutathione and glutathione reductase in chloroplasts:a proposed role in ascorbic acid metabolism. *Planta* 133 21–25. 10.1007/BF00386001 24425174

[B14] Fulda M. Schnurr J. Abbadi A. Heinz E. Browse J. (2004). Peroxisomal Acyl-CoA synthetase activity is essential for seedling development in *Arabidopsis thaliana*. *Plant Cell* 16 394–405. 10.1105/tpc.019646 14742880 PMC341912

[B15] Gémes K. Poór P. Horváth E. Kolbert Z. Szopkó’s D. Szepesi A. (2011). Cross-talk between salicylic acid and NaCl-generated reactive oxygen species and nitric oxide in tomato during acclimation to high salinity. *Physiol. Plant.* 142 179–192. 10.1111/j.1399-3054.2011.01461.x 21338371

[B16] Giannakoula A. Moustakas M. Syros T. Yupsanis T. (2010). Aluminium stress induces up-regulation of an efficient antioxidant system in the Al-tolerant maize line but not in the Al-sensitive line. *Environ. Exp. Bot.* 67 487–494. 10.1016/j.envexpbot.2009.07.010

[B17] Graham I. A. (2008). Seed storage oil mobilization. *Annu. Rev. Plant Biol.* 59 115–142. 10.1109/58.753032 18444898

[B18] Guo L. (2013). *Cloning and Expression of SDH Gene Related to Residues of Cucumis viride in Cucumber.* Dissertation. Northeast Agricultural University : Heilongjiang Sheng.

[B19] Hao J. H. Yi Y. Shang Q. M. Dong C. J. Zhang Z. (2012). Effect of exogenous salicylic acid on nitrogen assimilation of cucumber seedling under drought stress. *Acta Horticult. Sin.* 2012 81–90.

[B20] Houtkooper R. H. Cantó C. Wanders R. J. Auwerx J. (2010). The secret life ofNAD+: an old metabolite controlling new metabolic signaling pathways. *Endocr. Rev.* 31 194–223. 10.1210/er.2009-0026 20007326 PMC2852209

[B21] Lea U. S. Slimestad R. Smedvig P. (2007). Nitrogen deficiency enhances expression of specific MYB and bHLH transcription factors and accumulation of end products in the flavonoid pathway. *Planta* 225 1245–1253. 10.2307/23389788 17053893

[B22] Lescot M. Déhais P. Thijs G. Marchal K. Moreau Y. Van de Peer Y. (2002). PlantCARE, a database of plant cis-acting regulatory elements and a portal to tools for in silico analysis of promoter sequences. *Nucleic Acids Res.* 30 325–327. 10.1093/nar/30.1.325 11752327 PMC99092

[B23] Li L. (2013). *Subcellular Localization and Overexpression of Four OsVDAC Proteins in Rice and their Effects on Rice Growth.* Dissertation. South-central University for Nationalities: Wuhan Shi.

[B24] Li S. Qin Z. Xin M. Zhou X. (2016). Expression and functional analysis of Cswrky30 in cucumber under propamocarb stress. *Sci. Agric. Sin.* 2016 1277–1288.

[B25] Liu C. Qin Z. Zhou X. (2018). Expression and functional analysis of the propamocarb-related gene CsDIR16 in cucumbers. *BMC Plant Biol.* 18:16. 10.1186/s12870-018-1236-2 29347906 PMC5774166

[B26] Liu D. (2017). *Evaluation of Resistance of Main Cucumber Germplasm Resources.* Dissertation. Northeast Agricultural University:Heilongjiang Sheng.

[B27] Liu F. (2010). *Screening of Cucumber Germplasm Resources with Low Pesticide Residues.* Dissertation Northeast Agricultural University: Heilongjiang Sheng.

[B28] Ma B. (2010). Determination of remnant residues in cucumber fruits by gas chromatography. *J. Changjiang Veg.* 20 51–53.

[B29] Mazorra L. M. Nunez M. Hechavarria M. Coll F. Sánchez-Blanco M. J. (2002). Influence of brassinosteroids on antioxidant enzymes activity in tomato under different temperatures. *Plant Biol.* 45 593–596. 10.1023/a:1022390917656

[B30] Meng J. Qin Z. W. Zhou Z. Xin M. (2016). An Atp-binding cassette transporter gene from *Cucumis Sativus* L., Csabc19, is involved in propamocarb stress in *Arabidopsis Thaliana*. *Plant Mol. Biol. Rep.* 34 947–960. 10.1007/s11105-016-0976-0

[B31] Mittler R. (2002). Oxidative stress, antioxidants and stress tolerance. *Trends Plant Sci.* 7 405–410. 10.1016/S1360-1385(02)02312-9 12234732

[B32] NY/T761-2008. (2008). People’s Republic of China agricultural industry standard. Beijing: Ministry of Agriculture of the PRC.

[B33] Palmieri F. (2004). The mito ondrial transporter family SLC25. *Physiol. Pathol. Implica.* 447 689–709. 10.1007/s00424-003-1099-7 14598172

[B34] Palmieri L. Santoro A. Carrari F. Blanco E. Nunes-Nesi A. Arrigoni R. (2008). Identication and characterization of ADNT1, a novel mitochondrial adenine nucleotide transporterfrom *Arabidopsis*. *Plant Physiol.* 148 1797–1808. 10.1104/pp.108.130310 18923018 PMC2593658

[B35] Pollak N. Dölle C. Ziegler M. (2007). The power to reduce: pyridine nucleotides—small molecules with a multitude of functions. *Biochem. J.* 402 205–218. 10.1042/BJ20061638 17295611 PMC1798440

[B36] Schmittgen T. D. Livak K. J. (2008). Analyzing real-time PCR data by the comparative C(T) method. *Nat. Protoc.* 3 1101–1108. 10.1038/nprot.2008.73 18546601

[B37] Surassawade P. Ketsa S. van Dooran W. G. (2012). Salicylic acid alleviates chilling injury in anthurium (*Anthurium andraeanum* L.)Flowers. *Postharv. Biol. Technol.* 64 104–110. 10.1016/j.postharvbio.2011.10.002

[B38] Todisco S. Agrimi G. Castegna A. Palmieri F. (2006). Identication of themitochondrial NAD+transporter in Saccharomyces cerevisiae. *J. Biol. Chem.* 281 1524–1531. 10.1074/jbc.M510425200 16291748

[B39] Uimari A. (1997). Myb26:aMYB-like protein of pea flowers with affinity for promoter sofphenyl-propanoidgenes. *Plant J.* 12 1273–1284. 10.1046/j.1365-313x.1997.12061273.x 9450341

[B40] van Roermund C. W. Schroers M. G. Wiese J. Facchinelli F. Kurz S. Wilkinson S. (2016). The peroxisomal NAD carrier from arabidopsis imports NAD in exchange with AMP. *Plant Physiol.* 171 2127–2139. 10.1104/pp.16.00540 27208243 PMC4936582

[B41] Wan H. Zhao Z. Qian C. Sui Y. Malik A. A. Chen J. (2010). Selection of appropriate reference genes for gene expression studies by quantitative real-time polymerase chain reaction in cucumber. *Anal. Biochem.* 399 257–261. 10.1016/j.ab.2009.12.008 20005862

[B42] Wang W. (2018). Mitigation methods of herbicide damage in rice. *South China Agriculture* 16 42–44. 10.19415/j.cnki.1673-890x.2018.16.012

[B43] Wang Y. Zhou M. Lu Y. Yu A. Li J. (2017). Protective effect of 5-aminosalicylic acid on kidney of paraquat poisoned rats via Nrf 2-ARE signaling pathway. *Chin. Crit. Care Med.* 29 961–966. 10.3760/cma.j.issn.2095-4352.2017.11.001 29151408

[B44] Wu P. Zhou X. Wu T. Qin Z. Zhao W. Xin M. (2013). Transcriptome analysis reveals differentially expressed genesassociated with propamocarb response in cucumber(*Cucumis sativus* L.) Fruit. *Acta Physiol. Plant.* 35 2393–2406. 10.1007/s11738-013-1274-1

[B45] Wu R. Wang L. Wang Z. Shang H. Liu X. Zhu Y. (2009). Cloning and expression analysis of a dirigent protein gene from the resurrection plant boea hygrometrica. *Prog. Nat. Sci.* 19 347–352. 10.1016/j.pnsc.2008.07.010

[B46] Yoo S. D. Cho Y. H. Sheen J. (2007). *Arabidopsis* mesophyll protoplasts: a versatile cell system for transient gene expression analysis. *Nat. Protoc.* 2 1565–1572. 10.1038/nprot.2007.199 17585298

[B47] Zhang X. Zhang Z. Fan C. (2013). Causes and control measures of Cucumber Downy Mildew in Solar Greenhouse. *Modern Agric. Sci. Technol.* 2013:149.

[B48] Zhang Y. Zhang X. L. Liu B. Wang W. Liu X. Chen C. (2014). A GAMYB homologue CsGAMYB1 regulates sex expression of cucumber via anethylene- independent pathway. *J. Exp. Bot.* 65 3201–3213. 10.1093/jxb/eru176 24790111 PMC4071842

[B49] Zhao W. (2013). Comparative analysis of anatomical characteristics of pericarp of low pesticide residues in Cucumber. *China Veg.* 20 32–38.

