# Supplementary figures and images for: Expression and Functional Analysis of the Propamocarb-Related Gene CsMCF in Cucumber
Source: Front Plant Sci. 2019 Jul 4;10:871. doi: 10.3389/fpls.2019.00871 (PMC6620734; doi:10.3389/fpls.2019.00871)

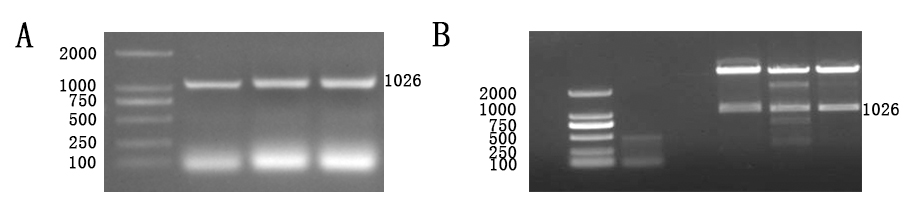

Supplement: FIGURE S1 — Cloning of CDS sequences of CsMCF and Sequence analysis. (A) Gel electrophoresis of PCR products. (B) Enzyme electrophoresis image. [file Image_1.JPEG]

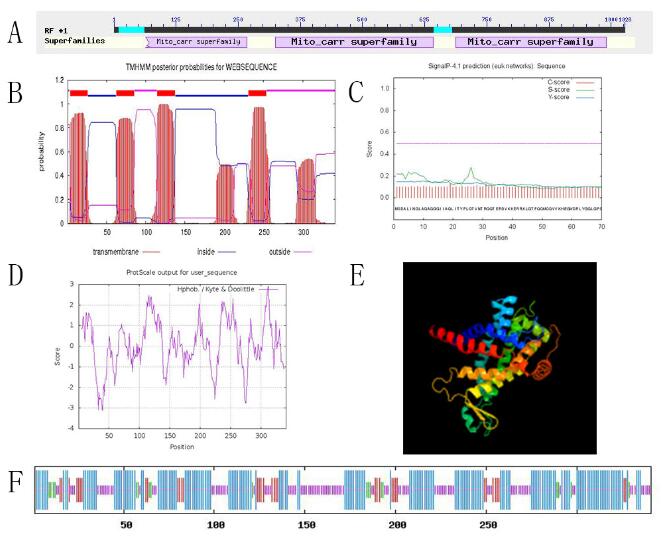

Supplement: FIGURE S2 — The Bioinformatics prediction of CsMCF coding protein. (A) The conserved domain of CsMCF coding protein. (B) Protein hydrophobicity prediction. (C) Signal peptide of CsMCF coding protein. (D) The transmembrane region of CsMCF coding protein. (E) Tertiary structure of protein. (F) Secondary protein structure. [file Image_2.JPEG]

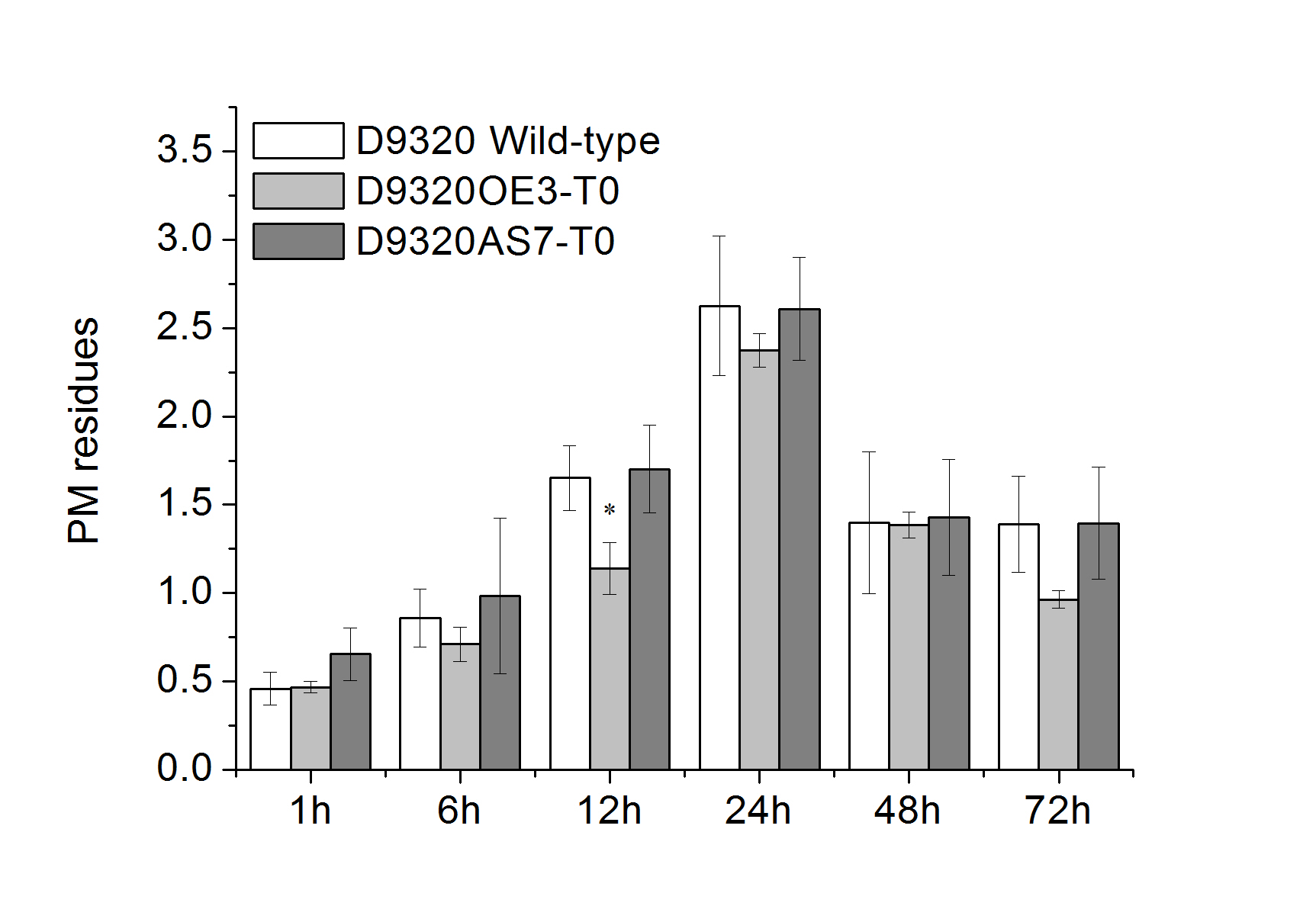

Supplement: FIGURE S3 — Detection of PM residues in T0 D9320OE3 and D9320AS7 lines plants. [file Image_3.JPEG]

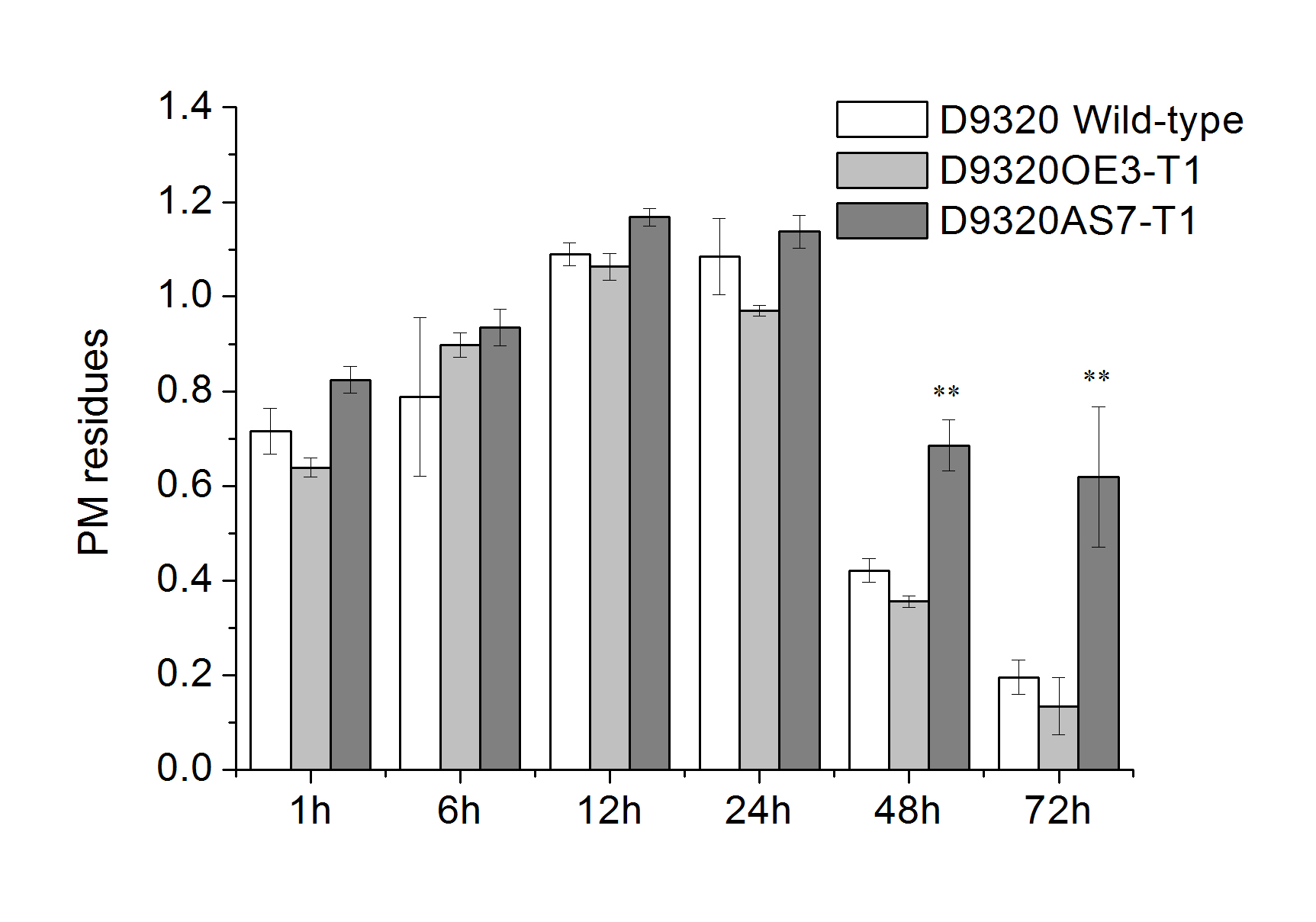

Supplement: FIGURE S4 — Detection of PM residues in T1 D9320OE3 and D9320AS7 lines plants. [file Image_4.JPEG]
