# Supplementary material for: Expression and Functional Analysis of the Propamocarb-Related Gene CsMCF in Cucumber
Source: Front Plant Sci. 2019 Jul 4;10:871. doi: 10.3389/fpls.2019.00871 (PMC6620734; doi:10.3389/fpls.2019.00871)
Supplement: TABLE S1 — All sequences in phylogenetic tree. [file Table_1.DOC]

| Name | | | | |  |  |  | Sequence |  |  |  |  |  |  |  |
| --- | --- | --- | --- | --- | --- | --- | --- | --- | --- | --- | --- | --- | --- | --- | --- |
| *CsMCF* | | | | |  | ATGTCGGACGCGTTGATCAATGGATTGGCCGGAGCTGGAGGAGGGATCATTGCCCAACTCATCACATACCCTCTTCAGAC | | | | | | | | | |
|  |  |  |  |  |  | TGTGAATACTCGTCAGCAAACAGAGCGCGATGTGAAGAAGGAGAGGAGAAAACTTGGAACATTTCAACAGATGTGTCAGG | | | | | | | | | |
|  |  |  |  |  |  | TTGTGAAACATGAAGGATGGGACCGGCTCTACGGTGGCTTGGGGCCGTCTCTGGTGGGTACGGCTGCATCTCAGGGTGTT | | | | | | | | | |
|  |  |  |  |  |  | TACTACTATTTCTACCAAATATTCAGGAACAAGGCTGAAGTTGCTTCTCTTGAACGAATGAAGGCAGGGATTGGAGATGG | | | | | | | | | |
|  |  |  |  |  |  | ATCTGTTGGGATGCTTTCCTCGCTTCTGGTGGCTGCTATATCTGGGTGTGTTAATGTACTGTTGACAAATCCTATATGGG | | | | | | | | | |
|  |  |  |  |  |  | TGGTGGTTACTCGGATGCAGACTCATAAAAAAATCTCAAAGCCGTCTCTACCTGGTGGAGCGCTGACTCCACTAGATGAA | | | | | | | | | |
|  |  |  |  |  |  | ACAATTCCGCCAACAGCAGTCGTTGATCCTCCTTCCTACGGAACCACTCATGCTATTCAAGAACTTTACGATGAAGCGGG | | | | | | | | | |
|  |  |  |  |  |  | AATCAAGGGTTTCTGGAAAGGAGTAATCCCAACAATGATCATGGTCAGCAACCCTTCTATACAGTACATGCTGTATGAAA | | | | | | | | | |
|  |  |  |  |  |  | CTTTGTTGAACAAATTGAAAAAACGACGTGCTTTACGGAAGGATGGGAGTGGAGTTACTGCATTGGAGATATTTTTTCTT | | | | | | | | | |
|  |  |  |  |  |  | GGTGCGTTGGCAAAACTTGGAGCGACTGTAGTAACCTATCCTCTTTTAGTTGTGAAGGCTAGGCTTCAAGCAAAACAAGT | | | | | | | | | |
|  |  |  |  |  |  | TGTAGCGGGAGACAAAAGACATCAGTATAAAGGCACATTGGACGCCATTCTCAAAATGATCCGATACGAAGGACTGTATG | | | | | | | | | |
|  | | | | |  | GATTTTACAAAGGGATGGGCACAAAAATCGTCCAGAGTGTTCTGGCTGCTGCTGTCTTGTTCATGGTTAAGGAAGAACTC | | | | | | | | | |
|  |  |  |  |  |  | GTACAAAGTGCTCGATTCCTTCTCACCAAGGGTCCCGTTGGCAGAATAAAATCAAAGCCTCAATGA | | | | | | | |  |  |
|  |  |  |  |  |  |  |  |  |  |  |  |  |  |  |  |
| *Momordica charantia* L. (XM 022301216.1) | | | | |  | TTTAATTTCTCTGGAAAATGATACGTAATTTCCCATCTTCAGGTTCAAGAATTAGCAAAACGCCATCGAAATGTCAATTT | | | | | | | | | |
|  |  |  |  |  |  | CATTTGGCTAGAACCGAAGTCGTAGACGCTATTTCTCTGCTCTCCGCGAATGAATTAGATCTCTTGTTTCGCTTCGATTT | | | | | | | | | |
|  |  |  |  |  |  | CCCCAGAAATTAACACCCATTTCGCTCCAATTTCTTTCTTCCGAATCACGATATACCCTTTTTCCCCCCTTTCTGTTACT | | | | | | | | | |
|  |  |  |  |  |  | GGGTTTTAGCGGGAAAGTATGTCGGACGCCTTGATCAATGGATTGTCCGGAGCTGGAGGAGGGATCATTGCTCAGCTCAT | | | | | | | | | |
|  |  |  |  |  |  | CACATACCCTCTTCAAACTGTGAATACCCGTCAGCAAACCGAGCGCGATGTGAAGAAGGAGAAGAGGAAACTTGGAACAT | | | | | | | | | |
|  |  |  |  |  |  | TTCAACAGATGTGCCAGGTTGTAAAACATGAAGGATGGGAGCGGCTCTACGGTGGCTTGGCGCCGTCTCTGGTGGGTACG | | | | | | | | | |
|  |  |  |  |  |  | GCTGCTTCTCAGGGTGTCTACTATTATTTCTACCAAATATTCAGGACCAAGGCTGAAGCTGCTTCTCTTGCACGAATGAA | | | | | | | | | |
|  |  |  |  |  |  | GGCAGGGGTTGGGGATGGATCAGTTGGGATGCTTTCCTCTCTTCTGGTGGCTGCCATATCTGGGTGTGTTAATGTGCTGT | | | | | | | | | |
|  |  |  |  |  |  | TGACAAATCCTATATGGGTTGTAGTTACACGAATGCAGACGCATAAAAAAATCTCAAAGCCGTCTCTGCCTGCTGGAGCA | | | | | | | | | |
|  |  |  |  |  |  | CCATTGACTCCTGTAGATGGAATAATTCAAACAGCAGTTGATCCTCCATCCTACAGAACCACTCATGCTATTCAAGAACT | | | | | | | | | |
|  |  |  |  |  |  | TTACGATGAAGCTGGAATCAGGGGTTTCTGGAAAGGAGTAATCCCAACATTGATCATGGTTAGCAATCCTTCTATACAGT | | | | | | | | | |
|  |  |  |  |  |  | ACATGCTGTATGAAACAATGTTGAGTAAATTGAAGAAACGACGTGCCTTACGCAAGGATGGCAATGGAGTTACTGCATTA | | | | | | | | | |
|  |  |  |  |  |  | GAGATATTTTTTCTCGGTGCCTTTGCAAAACTTGGAGCTACTGTAGTAACCTATCCTCTTTTAGTTGTGAAGGCTAGGCT | | | | | | | | | |
|  |  |  |  |  |  | TCAAGCGAAACAGATTGTAGCTGGAGACAAAAGACATCAATATAAAGGCACATTGGATGCCATCATCAAAATGATCCGAT | | | | | | | | | |
|  |  |  |  |  |  | ACGAAGGACTGTATGGATTTTACAAAGGGATGGGCACGAAAATCGTCCAGAGTGTTCTGGCTGCAGCTGTCTTGTTCATG | | | | | | | | | |
|  |  |  |  |  |  | GTCAAGGAAGAACTCGTCCGGGGCGTCCGGTTCCTTCTTGCGAAGGATGCAGCTGGTAGAATAAAATCAAAGCCTCAATG | | | | | | | | | |
|  |  |  |  |  |  | ATCCGGTGATTCCTATATGACTCATTACAGGTCTTTAAGAAAGTTATCCAAGCTTGCAATTTATCTCTTACTATCTTTCT | | | | | | | | | |
|  |  |  |  |  |  | TATACAGCTTAGTGCAGCATTCTTTACCAAACGTTTATAAAATTGTTAGGAAATTTAATTATGTCCTAGAAAAGATAGGT | | | | | | | | | |
|  |  |  |  |  |  | ATAAGATTTGGCCTGGAAATTGCCAATTGAAATATATCAATATGTTATAGCAGGAATATCTGATTCATGTGGAATCTTAT | | | | | | | | | |
|  |  |  |  |  |  | TGTACTTAGAGATTATTATATTGTTACTCTCAGATTAATAAATTTATGAGGATGACCATTACCCTCCCATTATGTA | | | | | | | | |  |
|  |  |  |  |  |  |  |  |  |  |  |  |  |  |  |  |
| Durio zibethinus Murr. (XM 022909305.1) | | | | |  | TTTGCTTTCTTAAACGGAACAGCGACGACCACTATAAAAGGAAAGAAACTGGCAGTTGGAATTTTCGATAGCGAACGCCG | | | | | | | | | |
|  |  |  |  |  |  | TCTCCCTCCACAAAATGGCCCTTTTTAATCTGTTTTACACGCTTCTTCGCTACGGCCAAAGTCAGAGATAGCATTTACTG | | | | | | | | | |
|  |  |  |  |  |  | CTTCTCTCTCTTCTATCTCTCTAAAACGTTAGATCTGTTTTCTACGTAACCAAACACCGGATTTTCTCGAGAAAATATTA | | | | | | | | | |
|  |  |  |  |  |  | GTTTTTTATTTCTTAGGGATTGAAACGATGTCGGACGCTTTGATCAATGGGCTGGCAGGAGCCGGTGGAGGGATCATAGC | | | | | | | | | |
|  |  |  |  |  |  | TCAACTCATCACGTATCCTCTTCAAACTGTCAATACTCGTCAACAAACAGAGCGTGATCTCAAGAGAGAGAAACGGAAAC | | | | | | | | | |
|  |  |  |  |  |  | TTGGAACGATTGAACAAATGTGCCAGGTTGTAAAACAAGAAGGATGGGCGCGGTTGTACGGCGGCTTAACGCCTTCTTTG | | | | | | | | | |
|  |  |  |  |  |  | GTTGGTACATCTGCATCTCAGGGTGTTTACTATTATTTCTATCAAATATTCAGGAACAAAGTTGAAACTACAGCACTTGA | | | | | | | | | |
|  |  |  |  |  |  | ACGCCATAAAAAAGGGATTGGAGATGGATCTGTTGGGATGCTTTCCTCACTTATTGTAGCTGCTTTAGCTGGGTGCGTAA | | | | | | | | | |
|  |  |  |  |  |  | ATGTGCTGTTGACAAACCCCATTTGGGTAGTTGTGACACGCATGCAGACTCACACAAAAACCTCAAAGAAAGACCATCCT | | | | | | | | | |
|  |  |  |  |  |  | GATAGGTTGCCAACTACTGCTCCAGAGGAAAAAGTTCTTTCTGTCATTGAGCCTCTTCCTTATGGAACTAGCCATGCGAT | | | | | | | | | |
|  |  |  |  |  |  | TCAAGAAGTCTATGATGAAGCTGGATTTTGGGGCTTCTGGAAAGGTGTATTCCCAACATTGATCATGGTGAGCAATCCTT | | | | | | | | | |
|  |  |  |  |  |  | CTATACAATTTATGCTATATGAAATGATGTTGAAGAATCTGAAGAAAAGACGATCTCGTAGTAAGAAGGGCAACAATGGA | | | | | | | | | |
|  |  |  |  |  |  | GTAACTGCTTTGGAGATTTTTCTTCTTGGAGCTCTGGCAAAACTTGGAGCTACTGTTGTGACATATCCTCTTCTCGTTGT | | | | | | | | | |
|  |  |  |  |  |  | AAAGTCAAGACTTCAAGCAAAACAAGTTACAACTGGGGACAGAAGGCATCATTACAAAGGGACTGTGGATGCTATTCTAA | | | | | | | | | |
|  |  |  |  |  |  | AGATGATCCGCTATGAAGGCTTCTCCGGCTTTTACAAAGGCATGAACACGAAAATCGTGCAAAGTGTTCTCGCAGCTGCT | | | | | | | | | |
|  |  |  |  |  |  | GTTTTATTCATGGTTAAAGAGGAACTCGTCAAGGGTGTTCGATTGTTGCTTGTTAAGGATGGCATCAACACGGTGAAATC | | | | | | | | | |
|  |  |  |  |  |  | AAAGCCTCCTTGACTGTGAAGCTGTCTCACTATCTCCCCACTCCGCCTCCTACTCCATTTGTTTCGACACTAACAAGTTG | | | | | | | | | |
|  |  |  |  |  |  | TTGGTCAAAATATTCAAACTTGGATTATAGTAGTATTTATCAGGCCATGTACCTTGAAATTAAAAGGATACAAAAAGATT | | | | | | | | | |
|  |  |  |  |  |  | ACTTGTTGAAAATAAAATCATTTACGGGTCCTATAAACAAGTTCAATTTATTCTC | | | | | | |  |  |  |
|  |  |  |  |  |  |  |  |  |  |  |  |  |  |  |  |
| *Rosa chinensis* Jacq. (XM 024321478.1) | | | | |  | CCATAAATGACTGCATTTAGTTGGAGAGGTTCGATAAGGCCAAAGTCCGAGATAGTCTTCTCCCAAAAGTTCAGATCTCT | | | | | | | | | |
|  |  |  |  |  |  | TTGATCCCTTGATGGCCGGAAAATAACAAACTTCCGTTCATTTTCTCCTGAAAATATGTCGGACGCCGTGATCAATGGCC | | | | | | | | | |
|  |  |  |  |  |  | TCGCCGGCGCCGGCGGAGGGATCATCGCCCAGCTCATCACATATCCACTCCAGACTGTGAACACTCGTCAGCAAACGGAA | | | | | | | | | |
|  |  |  |  |  |  | CGTGATCTGAAAAAGGAGAAGAGGAAGCTCGGAACTGTTGAACAAATAGGCCAGGTTATAAAGAATGAAGGATGGGAACG | | | | | | | | | |
|  |  |  |  |  |  | GTTGTACGGAGGTTTGACGCCGTCGTTGGTGGGAACAGCAGCATCTCAGGGTGTTTACTATTATTTCTATCAAATATTCA | | | | | | | | | |
|  |  |  |  |  |  | GGAACAAGGCTGAAGTTGCTGCCCTTGAACGTAGTAAGTTGGGCATTGGTGATGGATCTGTTGGAATGCTCTCTTCGCTA | | | | | | | | | |
|  |  |  |  |  |  | GTGGTGGCTGCATTATCTGGGTGTGTGAATGTACTATTGACAAATCCTATATGGGTAGTTGTTACACGCATGCAGACTCA | | | | | | | | | |
|  |  |  |  |  |  | TAGAAAAACTTCAAAGTCCCAGCCTGATCAGATGCTGTCAACTGATCCAGATGAAGCAATTGTTGCTGCAGCTGAGCCTC | | | | | | | | | |
|  |  |  |  |  |  | CTCCCTTCGGGACTAGTCATGCGGTACAAGAAGTTTATGATGAAGGTGGATTTTTCGGTTTCTGGAGAGGTGTATTTCCC | | | | | | | | | |
|  |  |  |  |  |  | ACATTGATCATGGTGAGTAATCCTTCCATGCAGTTTATGCTGTATGAAACTATGTTGAATAAGCTGAAGAAAAGACGTGC | | | | | | | | | |
|  |  |  |  |  |  | CTTGAGTAAGAAGAATAACAATGGTATTACTGCTGTAGAGATATTCCTGCTTGGTGCTTTGGCAAAACTAGGTGCTACTG | | | | | | | | | |
|  |  |  |  |  |  | TCGTGACATATCCTCTTTTAGTCGTGAAGGCGAGACTTCAAGCGAAACAGGTCACAACTGGTGACAAAAGGCATCATTAT | | | | | | | | | |
|  |  |  |  |  |  | AAAGGAACTCTGGATGCCATTTTAAAGATGATTCGCTATGAAGGGTTCTATGGTTTCTACAAAGGGATGAGCACAAAAAT | | | | | | | | | |
|  |  |  |  |  |  | AGTACAAAGTGTACTCGCCGCTGCTGTTTTGTTCATGGTCAAGGAAGAACTCGTCAGGGGTGTTCGGTTCATGCTCACTA | | | | | | | | | |
|  |  |  |  |  |  | ACAAAGCGAAATCAAAGCCTCCATAGATGGAAACCTCATTTAGGGGTGTTCTCATTCTCTGTCACACCCTTTCCCCCAAT | | | | | | | | | |
|  |  |  |  |  |  | ACACAATTCTTTGATTATCATAATATTTTCATGGCATGCATGCTCAAATGTATGTTTAGAAATGTATTGCCATTATCGCC | | | | | | | | | |
|  |  |  |  |  |  | GTATCGGCAAACTCAGATAACATACACACTAAGGGACTTTTGGTGGGGGGATCCATTTGAGGTAATGAGAATAGCGAATA | | | | | | | | | |
|  |  |  |  |  |  | ATTAGGCTTTTTTAAATCCAAGAAAACATTTCTAATGTTCTCA | | | | | |  |  |  |  |
|  |  |  |  |  |  |  |  |  |  |  |  |  |  |  |  |
| *Cucurbita pepo* L. (XM 023697586.1) | | | | |  | ATTACATGAACTAAAATTTGTAGAAAATGTTGCGTAATTTCCCACTCTTCAAGTCGTCAGGGTTTTCCAGATAGCGAACG | | | | | | | | | |
|  |  |  |  |  |  | TCATCGAAATGTATCATTTGGGTTCGATGCTCCGGTGCAGTTTCCCGGCAAACTAACAGGGCTTTTGCTTCAATTTCTAA | | | | | | | | | |
|  |  |  |  |  |  | CTTCAATTTCTCTTTTTGGGTTTTAGTGGGAAAGTATGTCGGACGCGTTGATCAATGGATTGGCTGGAGCTGGAGGAGGA | | | | | | | | | |
|  |  |  |  |  |  | ATCATTGCTCAGCTCATCACATACCCTCTTCAGACTGTGAATACTCGTCAGCAAACAGAGCGCGGCGTGAAGAAGGAAAA | | | | | | | | | |
|  |  |  |  |  |  | TAGGAAACCTGGAACGTTTCAACAGATGTGTCAGGTTGTAAAACATGAAGGATGGGACCGGCTCTACGGTGGCTTGGGGC | | | | | | | | | |
|  |  |  |  |  |  | CGTCTCTGGTTGGTACGGCTGCATCTCAGGGTGTTTACTATTATTTCTACCAAATATTCAGGAGCAAGGCTGAAGCTGCT | | | | | | | | | |
|  |  |  |  |  |  | GCTCTTGAACAGATGAAGGCAGGGATTGGAGACGGATCTGTTGGGATGCTTTCCTCGCTTCTGGTGGCTGCTATATCTGG | | | | | | | | | |
|  |  |  |  |  |  | GTGTGTCAATGTACTGTTGACAAATCCCATATGGGTGGTTGTTACTCGGATGCAGACACATAGAAAAGTCTCAAAGCCGT | | | | | | | | | |
|  |  |  |  |  |  | CTCTTCCCGGTGGAGCACCATTGACTCCACTAGATGAAACAATTCCCACAGCAGTCGGTCCTCCTTCATACGGAACCACT | | | | | | | | | |
|  |  |  |  |  |  | CATGCTATTCAGGAACTTTATGAGGAAGCTGGAATCAGGGGTTTCTGGAAAGGGGTAATCCCAACGATGATCATGGTTAG | | | | | | | | | |
|  |  |  |  |  |  | CAACCCTTCTATACAGTACATGCTGTATGAAACAATGTTGAACAAATTGAAGAAACGACGTGCCTTACGGAAAAATGGCA | | | | | | | | | |
|  |  |  |  |  |  | ATGGAGTTGCTGCACTAGAGATATTTTTTCTTGGTGCATTGGCAAAACTTGGAGCTACTGTAGTAACCTATCCCCTTCTA | | | | | | | | | |
|  |  |  |  |  |  | GTTGTGAAGTCTAGGCTTCAAGCAAAACAAGTCGTAGCTGGGGACAAAAGACATCAGTATAAAGGCACATTTGACGCCAT | | | | | | | | | |
|  |  |  |  |  |  | CATCAAAATGATCCGATACGAAGGAGTATACGGGTTTTACAAAGGAATGGGCACGAAGATCGTGCAGAGTGTTCTGGCTG | | | | | | | | | |
|  |  |  |  |  |  | CTGCTGTGTTGTTCATGGTTAAGGAAGAACTTGTCCGGAGTGCTCGATTCCTTCTCACCAAGAGTGCCGTTGGCAGAGTA | | | | | | | | | |
|  |  |  |  |  |  | AAACCAAAGCCTCAGTGACTCACTCCATTACAGGTTAATAAGAAGTTATCCAAGCCTCAATTTATGTCTATAATTTTAAT | | | | | | | | | |
|  |  |  |  |  |  | TATTATTCATCATCATCCTAGTGTAGTATTCTTATTTGAAGAAATTAATAGGAAATTTGTGTCCTAGAAAAATAGGCATA | | | | | | | | | |
|  |  |  |  |  |  | AGATTTGTGCTGGAAATTGTTGTTTAAAATATAACAGCAGGAACACTCAATTCATAGTCATAAACTGGAACGGCACATGT | | | | | | | | | |
|  |  |  |  |  |  | AAGATTTTATTGTCTTTACATATTATTATACAGTTACTCTCATATTAATATATGTATGATTCCAATGCTTTAATCA | | | | | | | | |  |
|  |  |  |  |  |  |  |  |  |  |  |  |  |  |  |  |
| *Ziziphus jujuba* Mill. var. *spinosa*  (XM 016021785.2) | | | | |  | TTCTCACTTTCTCTCTCTAAAAAAAAAGAAACTCTCTCTCTCTATCTTTCTCTCTATAATATTAGGTACTTTTTTATTTT | | | | | | | | | |
|  |  |  |  |  |  | TTATTTTTCCCTCTATGTCTGCCTATACATTCCCGAGAAAATAAGGAAAAACCATTTTCCCGAGAAAAAGAAAATGTCAG | | | | | | | | | |
|  |  |  |  |  |  | ACGCTTTGATCAATGGACTCGCCGGTGCTGGAGGAGGGATCATCGCTCAGCTTATTACTTATCCTCTTCAAACTGTGAAT | | | | | | | | | |
|  |  |  |  |  |  | ACTCGTCAACAGACGGAGCGTGATCTTAAGAAGGAGAAGAGGAAGCTTGGAACTATTCAACAAATGTGTCAGGTTGTGAA | | | | | | | | | |
|  |  |  |  |  |  | ACAAGAAGGATGGGGAAGATTATACGGAGGTTTAACGCCATCGTTAGCAGGCACAGCTGCTTCTCAAGGTGTTTACTATT | | | | | | | | | |
|  |  |  |  |  |  | ATTTCTATCAAATATTCAGGAACAAGGCTGAAGTTGCTGCACTTGAACGAAGGAAGTTAGGAATAGGTGATGGATCAGTT | | | | | | | | | |
|  |  |  |  |  |  | GGAATGTTCACCTCACTTCTGGTGGCTGCTTTGTCAGGGTGTGTGAATGTGCTGTTGACAAACCCTATATGGGTAGTTGT | | | | | | | | | |
|  |  |  |  |  |  | TACCCGCATGCAGACTCATACAAAAATCTCAACGAAGGCCACGCCTGGTGAGACACCATTGATTACTGCAGATGAAGCCG | | | | | | | | | |
|  |  |  |  |  |  | TTCTTGCTGCAGCAGCTGAGCCTCCTCCCTATGGGACTGCTCATGTGGTCCAGGAAGTTTTTGATGAAGCTGGAATTTGG | | | | | | | | | |
|  |  |  |  |  |  | GGTTTCTGGAAAGGTGTATTACCGACATTGATTATGGTTAGTAATCCTTCCATACAATTCATGCTTTATGAAACCATGTT | | | | | | | | | |
|  |  |  |  |  |  | GAAGAAGCTGAAGCAAAGCCGTGCCTCGAATAAGAATGGTAACAGTGTGGTTACTGCTTTAGAGATATTTCTTCTCGGTG | | | | | | | | | |
|  |  |  |  |  |  | CTCTGGCAAAACTAGGGGCTACCGTTGTGACATATCCTCTTTTAGTTGTGAAGTCGAGGCTTCAAGCAAAACAACTTACA | | | | | | | | | |
|  |  |  |  |  |  | ACTGGTGACAAGAGGCATCATTACAAAGGCACATTGGATGCTATTTTGAAGATGATTCACCATGAAGGATTTTATGGGTT | | | | | | | | | |
|  |  |  |  |  |  | TTACAAAGGGATGAGAACCAAAATTGTACAGAGTGTTCTTGCTGCTGCTGTACTGTTCATGGTCAAGGAAGAACTTGTTC | | | | | | | | | |
|  |  |  |  |  |  | AAGGTGCTCGGTTTTTGCTTACCAAGAATGCCATTAACACAGTGAAATCAAAGCCATCATGATTACAACTCTTAGTTCAT | | | | | | | | | |
|  |  |  |  |  |  | TTCATTCATATATGATACGATCTTACTAGAAATTCTATTGTCAGTTGCTTGCTTATTATGTGATTCAGCATTTGCAATTG | | | | | | | | | |
|  |  |  |  |  |  | GCAAACTCGGACCCCAAAATAAAATAGGATGTAAGGGGATAAATTGAAAGCAAAATGATTGTTATACTTGTACCTCTTCT | | | | | | | | | |
|  |  |  |  |  |  | GTTAGTGGATTTTTGTGAAAACGTATTTTTTTAGTGGGATTTTCTTTTTTCTTTT | | | | | | |  |  |  |
|  |  |  |  |  |  |  |  |  |  |  |  |  |  |  |  |
| *Glycine max* (Linn.) Merr. (XM 006591315.2) | | | | |  | GAGCCTTACAAGTTAAGCAGAGTCAAGGGTAGAATGGTCTTTAAATATTTTCATAATTTTGACATTTTCTTTTCCCAAAC | | | | | | | | | |
|  |  |  |  |  |  | GTAACAACCAAAGTGAAGCCACCTTGTTGTGCTGAGTCGTGTCGTGGGGGTACAGTACATCGCAGCATTCACTCTCATTG | | | | | | | | | |
|  |  |  |  |  |  | ATTGAACTAAATCCAAGATCATATTTTTATTTTCAATTCCCAACCGCCATTACTACAACATAGACTAGTGCATAGTGCAT | | | | | | | | | |
|  |  |  |  |  |  | AATAGGCAGTGCGGTGCATTCCCTTGTTTACCATGCTTTCCTGAGAGAGAACACTAAAACCCCTCAAATCCTCAATACAA | | | | | | | | | |
|  |  |  |  |  |  | ATAGATTTTCATTCCTCCGAGGGAAAAATAAATGTCGGACGCTTTGATCAATGGATTGGCCGGAGCTGGAGGAGGGATCA | | | | | | | | | |
|  |  |  |  |  |  | TTGCTCAGCTCATCACTTACCCACTTCAAACTGTAAATACTCGTCAACAAACCGAGCGTGATCCGAAGAAAGACACGAGG | | | | | | | | | |
|  |  |  |  |  |  | AGTCAAGGGACCCTTGAACGAATGTGCCAGGTTGTAAAAGAAGAGGGGTGGGAACGGTTGTATGGAGGCTTGATGCCATC | | | | | | | | | |
|  |  |  |  |  |  | GGTAGTGGGTACAGCTGCGTCTCAGGGTGTTTACTATTATCTCTATCAAATATTCAGGAACAAAGCTGAAGCAGCTGCAC | | | | | | | | | |
|  |  |  |  |  |  | TACAACAAAAGAAAATGGGCGTCGGCGATGGATCAGTTGGGATGCTCTCCTCACTTGTTGTTGCTGTTTTATCTGGGTCT | | | | | | | | | |
|  |  |  |  |  |  | GTTACCGTGCTGTTGACAAATCCCATATGGGTAGTTGCTACGCGTATGCAGACACATAGAAAAGAGTTGAACAGAACTCC | | | | | | | | | |
|  |  |  |  |  |  | AGCTGATCAGGGTTTGTTAGTTTCCACTGAGCAGCCAATTCTTTCTGCAGTTGAGCATCTTCCTTATGGAACTAGTCAGG | | | | | | | | | |
|  |  |  |  |  |  | TGATTCAAGATATCTACAGTGAAGCTGGAATTTTGGGTTTCTGGAAAGGTGTATTACCAACATTAATCATGGTAAGCAAT | | | | | | | | | |
|  |  |  |  |  |  | CCTTCCATACAGTTCATGCTGTATGAAGCCATGTTGGTGAAGTTAAGAAAAAGACGTGCTTGGAGTAAGAAGGGTAGCAA | | | | | | | | | |
|  |  |  |  |  |  | TGGGGTAACTGCTTTAGAGATATTTCTTATTGGAGCTTTAGCTAAGCTTGGAGCTACTGTTGTAACCTATCCGATCCTAG | | | | | | | | | |
|  |  |  |  |  |  | TTGTGAAGGCAAGGCTCCAAGCTAGACAGGACAAAACTGGAGACAAGAGGCACCATTACAAAGGTACATGGGATGCTATT | | | | | | | | | |
|  |  |  |  |  |  | ATAAAAATGATCCGTTATGAAGGGTTTAATGGGTTTTACAATGGTATGGGCACAAAAATTGTACAAAGTGTGCTGGCTGC | | | | | | | | | |
|  |  |  |  |  |  | TGCTGTTTTGTTCATGATGAAGGAAGAACTAGTTAGGGGGGTTCGTTTCTTGCTTGCCAATGATGCTGTAAAGCCAAAGC | | | | | | | | | |
|  |  |  |  |  |  | ATCCGTGATTGAAGAGGCCACACTAGCTTGATCAAGATTTCATGATGTACCATCTTGAGGTAGCAAATTAGTTCAAATGG | | | | | | | | | |
|  |  |  |  |  |  | TAGGGGAATTAGAGTGATTCAAACAAAATATTAAGATATATGGCTGTAACTGTATCTTTGATTATTTTCATCAGGTTAGT | | | | | | | | | |
|  |  |  |  |  |  | TTCCATAGAGGAAATTCATGCCAAAAATTACATGCTGGATTCATTGGTTCGTTTCATGAACAAAACAAGGTTGTAGATTT | | | | | | | | | |
|  |  |  |  |  |  | TGAGTATGCTGCTGAAACAAATATTCTTTTTTACGAATGAATATCACTATCTGATTTCATTGGTAGCTAAA | | | | | | | | |  |
|  |  |  |  |  |  |  |  |  |  |  |  |  |  |  |  |
| *Beta vulgaris* L. (XM 010691750.2) | | | | |  | TGGGGCACTTTACCCAAGACGATATACACTTTTACTTGAACGCAACAAACACGCGCGCTCTCTCTCTCTCTCCTCCAATC | | | | | | | | | |
|  |  |  |  |  |  | TCCATCCAAAATTATAGAATTGACGCAGAAATGCAACTACCGAAAGCGAACTTCATTTCGCTACATCCAAAGTCAAGATC | | | | | | | | | |
|  |  |  |  |  |  | TTAGTCCTTCTTCAGTCCCCCATTTTTTCTTCTCTTCTCTTCTCACCTGATCAATCAATAATCTCTTTCTCTCCGATCAT | | | | | | | | | |
|  |  |  |  |  |  | AATTTCTCGATCATGTCTGATGCTCTCATCAATGGCCTCGCCGGCGCCGGCGGTGGTATCATCGCTCAACTCATCACTTA | | | | | | | | | |
|  |  |  |  |  |  | TCCCTTACAAACTGTTAATACTCGGCAGCAAACGGATCGAGATCCTAACAACGCGAAGACTAAAATTGGAACTCTTGAAC | | | | | | | | | |
|  |  |  |  |  |  | AAATGTGTCAGGTTGTGAAACATGAAGGATGGGGGAGACTGTATGGAGGATTAACTCCGTCTTTAGTTGGTACTGCCGCT | | | | | | | | | |
|  |  |  |  |  |  | TCTCAGGGTGTTTATTATTATTTCTATCAAATATTCAGGAACAAGGCTGAAGGTATTGCACTTGAACGCAGGAAGAGAGG | | | | | | | | | |
|  |  |  |  |  |  | GCTTGGGGATGGCTCAGTTGGCATGTTCTCATCATTGGTCGTGGCGGCTATGTCTGGGTGTACGAATGTGTTGTTGACAA | | | | | | | | | |
|  |  |  |  |  |  | ATCCCATATGGTTAGTTGTAACCCGCATGCAGACGCATACTAAGGTTTCCAAGAAAGGACATCCCAGTCAAGCTATAAGA | | | | | | | | | |
|  |  |  |  |  |  | TCTTCTGAAGACAATCTAGTAGCAATTGAGCCACCTGCGTATGGAACTAGCAATGCAATACAAGAAGTGTTCGATGAAGC | | | | | | | | | |
|  |  |  |  |  |  | TGGGTTCTTAGGCTTCTGGAAAGGCGTGGTTCCAACATTGATTATGGTAAGTAATCCCTCCATACAGTTCATGCTGTATG | | | | | | | | | |
|  |  |  |  |  |  | AGACCCTATTGAAAAAGCTGAGGCAGAGGCGCGCGATGAACAAAAATGGTAGCAATGGAGTCACTGCTCTAGAGATATTT | | | | | | | | | |
|  |  |  |  |  |  | TTACTCGGGGCTCTAGCAAAGCTTGGAGCTACTGTCGTGACATACCCTCTTTTGGTGGTGAAGTCAAGGCTTCAAGCAAA | | | | | | | | | |
|  |  |  |  |  |  | GCAAGTTGCTGGCACAGACAAGAAGCATCACTATAAAGGTACCGTTGACGCAATCATGAAGATAATTCATTTTGAGGGTT | | | | | | | | | |
|  |  |  |  |  |  | TGTACGGATTTTACAAAGGGATGAGCACAAAGATTGTACAAAGTGTTCTTGGAGCTGCTATTCTTTTTATGATTAAGGAG | | | | | | | | | |
|  |  |  |  |  |  | GAACTTGTGAAAGGAGCACGCCTATTACTTTCAAGAAATGATTCTTTGTTAAAGGCTCGTCAATTATAGGTTTGATTCTT | | | | | | | | | |
|  |  |  |  |  |  | TTTCACCATATAAGTGTATCTTTCTGGTGGCAAATAAAAAAGTGAAGTCAAGTTCATTGTTGAGCTGAGCGAAAGTATGG | | | | | | | | | |
|  |  |  |  |  |  | CTTTAACCATGAGATGTACTCCAATGGAGGATGCAAGTCTTCAGAAGTGCAGTTTCTCTCTTTAATGGCCGACTATTCTG | | | | | | | | | |
|  |  |  |  |  |  | TTTAACCAATATGGCAAACAGGCTATTACTCGTAGAAGTGTTTTAAATTTAGATGGCAGGGATTATCGAAAATGATTCGT | | | | | | | | | |
|  |  |  |  |  |  | GTTCCTTGCCATTGTCGACGCCAATTCTAAATTGCTGATCACTGGAGTAGCAAGACGATGACCTCGGTGCCAAAATCTGC | | | | | | | | | |
|  |  |  |  |  |  | TGTTCCTCTACCTTATCTGAAAGCAAAACTCAAAAGTTACAATCGTGATTCGTGTATGTAATGTGAATCCTTCTTAACTT | | | | | | | | | |
|  |  |  |  |  |  | TGTCTTAGTCGCTTCATAGAACTTTGATTGTAATTAAAGACACGGTCTATAGTTTCCATTATGATTTCTGATTACTCCAT | | | | | | | | | |
|  |  |  |  |  |  | ATATCGTCAATTGACTCCTCTCATTACTTCAATA | | | |  |  |  |  |  |  |
|  |  |  |  |  |  |  |  |  |  |  |  |  |  |  |  |
| *Cucumis melo* L. (XM 008464998.2 ) | | | | |  | ACATTTTTCTCATTAATTTGATAGAAAATGATGCGTAATTTCCCATCTTCAAGCCGTTAGTCAGAATTTTCCCAGATATC | | | | | | | | | |
|  |  |  |  |  |  | AAACGCCATCGAAAATGTCAATATCATTTGGGTAGAAGTTAGAACAGAGGCAAAGTCGTACATGGATTTCCTTCTCCGAT | | | | | | | | | |
|  |  |  |  |  |  | TGAATTTGAGCTAGATCTCTTTCTTTTCACGTCAATGCTCCGGTTCCGGTGCATTTTCCCGGTGAACTAACACCGTTTTT | | | | | | | | | |
|  |  |  |  |  |  | TCCTTCTCTTTCTATCTTCCCACATACCCCCTTTTTCTTCCTTCTTCTTTGCTGTCTCTAATTGGGTTTTTAGCTGGAAA | | | | | | | | | |
|  |  |  |  |  |  | GTATGTCGGACGCGTTGATCAATGGATTGGCCGGAGCTGGAGGAGGGATCATTGCTCAACTCATCACATACCCTCTTCAG | | | | | | | | | |
|  |  |  |  |  |  | ACTGTGAATACTCGTCAGCAAACAGAGCGCGATGTGAAGAAGGAGAAGAGAAAACTTGGAACATTTCAACAGATGTGTCA | | | | | | | | | |
|  |  |  |  |  |  | GGTTGTGAAACATGAAGGATGGGACCGGCTCTACGGTGGCTTGGGGCCGTCTCTGGTGGGTACGGCTGCATCTCAGGGCG | | | | | | | | | |
|  |  |  |  |  |  | TTTACTATTATTTCTACCAAATATTCAGGAACAAGGCTGAAGTTGCTTCACTTGAACGAATGAAGGCAGGGATTGGAGAT | | | | | | | | | |
|  |  |  |  |  |  | GGATCTGTTGGGATGCTTTCCTCACTTCTGGTGGCTGCTATATCTGGGTGTGTTAATGTACTGTTGACAAATCCTATATG | | | | | | | | | |
|  |  |  |  |  |  | GGTGGTTGTTACTCGGATGCAGACACATAAAAAAGTCTCAAAGCCGTCTCTACCTGGTCGAGCGCTGACTCCACTAGATG | | | | | | | | | |
|  |  |  |  |  |  | AAACAATTCCGCCGACAGCAGTTGTTGATCCTCCTTCCTACGGAACCACTCATGCTATTCAAGAACTTTATGATGAAGCG | | | | | | | | | |
|  |  |  |  |  |  | GGAATCAAGGGTTTCTGGAAAGGGGTAATCCCAACAATGATCATGGTCAGCAACCCTTCTATACAATACATGCTGTATGA | | | | | | | | | |
|  |  |  |  |  |  | AACTTTGTTGAACAAGTTGAAAAAACGACGTGCTTTACGGAAGGATGGGAGTGGAGTTACTGCATTGGAGATATTTTTTC | | | | | | | | | |
|  |  |  |  |  |  | TTGGTGCGGTTGCAAAACTTGGAGCAACGGTAGTAACCTATCCTCTTTTAGTTGTGAAGGCTAGGCTTCAAGCAAAACAA | | | | | | | | | |
|  |  |  |  |  |  | GCCGTAGCGGGAGACAAAAGACATCAGTATAAAGGCACATTGGACGCCATTCTCAAAATGATCCGATACGAAGGACTGTA | | | | | | | | | |
|  |  |  |  |  |  | TGGATTTTACAAAGGGATGGGCACGAAAATCGTCCAGAGTGTTCTGGCTGCTGCTGTCTTGTTCATGGTTAAGGAAGAAC | | | | | | | | | |
|  |  |  |  |  |  | TCGTCCGAAGTGCTCGATTCCTTCTCACCAAGGGTCCCGTTGGCAGAATAAAATCAAAGCCTCAATGATCTGATAAAATG | | | | | | | | | |
|  |  |  |  |  |  | CTTATATGACCCCATTACAGGTTTCTTAAGAAGTTATCCTAGCCAGAAATTTATGTTATTCATCCTAGTGCAGTATTCTT | | | | | | | | | |
|  |  |  |  |  |  | GACCAAACCCTTATTTGAAATTATTAGGAAAATTTGTGTCCAAGAAAAGAAATAAGATTAAGATTTGGGCTGGAAATTGT | | | | | | | | | |
|  |  |  |  |  |  | TGTTTGAAATATAACAATATGCTATAGCAGAAATATTGGATTCATAGTTATAAACTGGAGTGGCCTATGTTTTAGGTCAA | | | | | | | | | |
|  |  |  |  |  |  | TTATACCATTTAGAGATTATTATATAGTCACAACCATATTACAAAAAAAAATATTCTTAAAACCA | | | | | | | |  |  |
|  |  |  |  |  |  |  |  |  |  |  |  |  |  |  |  |
| *Abrus precatorius*  L. (XM 017561667.1) | | | | |  | ATTTTGACATTTTCTTTCCCAGACGCAACAACCAAAGTGAAACTTTGTAGTTGAGTCGAATCGTGTCGTGGTGGACATCA | | | | | | | | | |
|  |  |  |  |  |  | CATTCATTTTCATTGATTGGTACCAATCCAACACCATTTTTCATGTGCCAACGGCGAGATCGCCATTACTACAACACAGA | | | | | | | | | |
|  |  |  |  |  |  | CAAGAAGTAGCAAACACGAGGAGGGAGTGGGTGCATTTCCTGCTTCTTCCCTGAGACCGCAGTTAAAGTGTAAACCCCTT | | | | | | | | | |
|  |  |  |  |  |  | AACACTAAAACCCAGATTTTCGTTTCCCCCCCAGTGGGGAAAACAGAAAAAAAAATAGAGGAAGAAAAACATGTCGGACG | | | | | | | | | |
|  |  |  |  |  |  | CTTTGATCAATGGATTGGCCGGAGCTGGAGGCGGGATCATTGCTCAGCTCATCACGTACCCACTTTCAACTGTAAACACT | | | | | | | | | |
|  |  |  |  |  |  | CGTCAACAAACCGAGCGTGATCCGAAGAGAGAGACAAGCAGCCAAGGTGCCCTCGAACGAATGTGCCAGGTTGTAAAAGA | | | | | | | | | |
|  |  |  |  |  |  | AGAAGGGTGGGAACGGTTGTATGGAGGCTTGATGCCGTCTCTAGTGGGTACCGCTGCGTCTCAGGGTGTTTACTATTATT | | | | | | | | | |
|  |  |  |  |  |  | TCTATCAAATATTCAGGAACATGGCTGAAGCAGCTGCGCTACAGAAAAAGAAAATGGGTGTCAGTGATGGATCAGTTGGG | | | | | | | | | |
|  |  |  |  |  |  | ATGCTCTCCTCTCTTCTCACTGCTGCTTTATCCGGGTGTGTTAACGTGCTCTTGACAAATCCCATATGGGTAGTTGTTAC | | | | | | | | | |
|  |  |  |  |  |  | CCGTATGCAGGCACATAGAAAAGAGTCGAACCATGCACCGGATCAAGGTTTGTTAGTTGCCACTGAGCAAGAAACTATTT | | | | | | | | | |
|  |  |  |  |  |  | CTGCAGTTGAGCCTCTTCCTTACGGAACTAGCCGTGTGATTCAAGAAATCTTTGATGAAGCTGGTATTCGGGGTTTCTGG | | | | | | | | | |
|  |  |  |  |  |  | AAAGGTGTATTACCATCACTGATCATGGTAAGCAATCCTTCCATACAGTTCATGCTGTATGAAGCCATGTTGTCAAAGTT | | | | | | | | | |
|  |  |  |  |  |  | AAGAAAAAGACGTAACAGCAACAGGGTAACTGCGTTAGAGATATTTTTTCTTGGGGCTTTGGCTAAGCTTGGAGCTACTG | | | | | | | | | |
|  |  |  |  |  |  | TTGTAACTTATCCACTCCTTGTTGTAAAGGCGAGGCTCCAGGCTAGGCAGGATAAAACAGGAGACAGGAGGCACCATTAC | | | | | | | | | |
|  |  |  |  |  |  | AAAGGTACCAGGGATGCTATTATTAAAATGATCCGCTATGAAGGGTTGGATGGGTTTTACAAAGGTATGGGCACAAAAAT | | | | | | | | | |
|  |  |  |  |  |  | TGTACAGAGTGTGCTTGCTGCTGCTGTTATATTCACCATGAAAGAAGAACTGGTTAGGGGGGTTCACTTCTTGCTTGCCA | | | | | | | | | |
|  |  |  |  |  |  | GGCAAGCTGCCAAAACTGTAAAGCTAAAGCCTGTCTGATTGATTCGAGGTCGCTGGCCTCTTCAATATTTCGTTTACCAT | | | | | | | | | |
|  |  |  |  |  |  | CTTCAGTTAGCAAATTAGTTTTAAAATGGTAGTTGGAGAACAAAACTGATTCAAACAATATGTTAAAATATCTGCATCTT | | | | | | | | | |
|  |  |  |  |  |  | CATTATTTTTATCACAAAGCTTTGGTTCATTTTATGAACAAAGCAGGCTTGTGGGTTTGAA | | | | | | | |  |  |
|  |  |  |  |  |  |  |  |  |  |  |  |  |  |  |  |
| *Phaseolus vulgaris* (XM 007163454.1) | | | | |  | GAGTCGAATCGTGTCGTGTGGGACATCACATTCATTTTCATTCATTGGTACAATCCAACGCCATTTTTCATGTGCCAACG | | | | | | | | | |
|  |  |  |  |  |  | CCGAGATCGCCATTACTACAACACAGACAAGTACCAAACACCACGAAGGAGTGGGTGCATTCCCTGCTTCTTCACTCAGA | | | | | | | | | |
|  |  |  |  |  |  | CAACAGTTAAAGTGTAAAATCCCTTAATACTACAACCCAGATTTTCGTTCCCCCCAGTGGGGAAAACAGAAAAAAATAAA | | | | | | | | | |
|  |  |  |  |  |  | GGAAGAAAAACATGTCGGAGGCTTTGATCAATGGATTGGCCGGAGCTGGAGGAGGGATCATTGCTCAGCTCATCACATAC | | | | | | | | | |
|  |  |  |  |  |  | CCACTTTCAACTGTAAACACTCGTCAACAAACCGAGCGTGATCCGAAGAGAGCGACGAGGAATCAAGGTGCCCTCGAACG | | | | | | | | | |
|  |  |  |  |  |  | AATGTGCAAGGTTGTAAAAGAAGAAGGGTGGGGACGATTGTATGGAGGCTTGACGCCATCTCTAGTGGGTACCGCTGCGT | | | | | | | | | |
|  |  |  |  |  |  | CTCAGGGTGTTTACTATTATTTCTATCAAATATTCAGGAACATGGCTGCAGAAGCTGCGGTACAGAAAAAGAAAAAGGGT | | | | | | | | | |
|  |  |  |  |  |  | GTCGGTGATGGATCAATTGGGATGCTCTCCTCTCTCCTCACTGCTGCTTTATCCGGGTGTGTTAACGTGCTCTTGACAAA | | | | | | | | | |
|  |  |  |  |  |  | TCCCATTTGGGTAGTTGTTACCCGTATGCAGGCACATAGAAAAGGGTCGAACCATGCACCTGATGAGGGTTTGTTAGTTG | | | | | | | | | |
|  |  |  |  |  |  | CCACTGAGCAAGCAATTATTCCTGCAGTTGAACCTCTTCCTTATGGAACTAGCCATGTGATCGAAGAAATCTTTGATGAA | | | | | | | | | |
|  |  |  |  |  |  | GCTGGTATTCGGGGTTTCTGGAAAGGTGTATTACCATCATTGATCATGGTAAGCAATCCTTCCATGCAATTCATGCTGTA | | | | | | | | | |
|  |  |  |  |  |  | TGAAGCCATGTTGTCAAAGTTAAGAAAAAGAAATAATAGCAACAGGGTAACTGCGTTACAGATATTTCTTCTTGGAGCTT | | | | | | | | | |
|  |  |  |  |  |  | TGGCTAAGCTTGGAGCTACTGTTGTAACTTATCCTCTTCTAGTTGTAAAGGCGAGGCTCCAGGCTAGACAGGATAAAACA | | | | | | | | | |
|  |  |  |  |  |  | GGAGACAGGAGACAGCATTACAAAGGTACTTGGGATGCTATTATTAAAATGATTCGCTATGAGGGGTTGAATGGGTTTTA | | | | | | | | | |
|  |  |  |  |  |  | CAAAGGTATGGGAACAAAAATTGTACAAAGTGTGCTTGCTGCTGCTGTTATATTCACTATAAAAGAAGAACTGGTTAGGG | | | | | | | | | |
|  |  |  |  |  |  | GGGTTCACTTCTTGCTTGCCAAGGAAGCTGCCAAAACTGTAAAGCAAAAGCGTGTGTGATTGATTGGAGGCCACAAGCTT | | | | | | | | | |
|  |  |  |  |  |  | GATCAAGATTTCGTTTACCATCTTCAGTTAGCAAATTAGTTAAAATGGTAGTAGGGGAACTAAAGTGATTCAAACAATAT | | | | | | | | | |
|  |  |  |  |  |  | ATTGATTATTTTTATCAGGTAGTTTCCTTATAGCAAATTCATGTCATGATCATACACTGGATTCATTGATTCATTCCATG | | | | | | | | | |
|  |  |  |  |  |  | AACAAAGCAGGGTTTTAGGTTTGAAGTATGCTGCTGGAATGGAACAGAAATTCATTTTACTAAT | | | | | | | |  |  |
|  |  |  |  |  |  |  |  |  |  |  |  |  |  |  |  |
| *Cucurbita moschata* (023140220.1) | | | | |  | CCCATTAATTAAAACCTGCCCTTTCCCTCAATTTATTAGAAAATAATGCGTTATTTCCCATCTTCAAGTCGTCGAGATTT | | | | | | | | | |
|  |  |  |  |  |  | TCTCACATAGCTAAGGCCATCGAAATGTCAATTTCATTCGGGTAGAACACAGCCAAAGTCGTACATGGTTCCTCTCCAAA | | | | | | | | | |
|  |  |  |  |  |  | TAAGTTTGACGGGAAAGTATGTCGGATGCGTTGATCAATGGATTGGCAGGAGCTGGAGGAGGGGTCATTGCTCAACTCAT | | | | | | | | | |
|  |  |  |  |  |  | CACATACCCTCTTCAAACTGTGAATACTCGGCAGCAAACAGAACGCGATGTGAAGAACGAGAAGAGGAAACTTGGAACAT | | | | | | | | | |
|  |  |  |  |  |  | TGCAGCTCATGTGTCAGGTTGTAAAACATGAAGGATGGGACCGGCTGTATGGTGGCTTGGGGCCGTCTCTGGTGGGTACG | | | | | | | | | |
|  |  |  |  |  |  | TCCGTGTCTCAGGGTGTTTACTATTATTTCTACCAAATATTCAGGAGCAAGGCTGAAGCTGCTTCTCTTGAACGAATGAA | | | | | | | | | |
|  |  |  |  |  |  | GGCGGGGATTGGAGACGGATCAGTTGGGATGCTTTCCTCTCTTCTGGTGGCTGCTGTATCTGGGTGTGTTAACGTACTGT | | | | | | | | | |
|  |  |  |  |  |  | TGACAAATCCGATATGGGTGGTTGTTACACGGATGCAGACACATAAAAAGGTCTCAAAGCCGTCTCTACCTGGTGGAGCA | | | | | | | | | |
|  |  |  |  |  |  | CCTTTGACTCCTCTGGATGAAACTGTTCCGGCAGCAGTCAATCCTCCTTCCTACAGAACCACTCATGCTATTCAGGAGCT | | | | | | | | | |
|  |  |  |  |  |  | TTATGATGAAGCTGGAATCAGGGGTTTCTGGAAAGGGGTAATCCCAACATTGATCATGGTTAGCAACCCTTCTATACAGT | | | | | | | | | |
|  |  |  |  |  |  | TCATGCTGTATGAAACAATGTTGAACAAGTTGAAGAAACAACGCGCCTTACGTAAAGTTGGCAGTGGAGTTACTGCATTG | | | | | | | | | |
|  |  |  |  |  |  | GAGGTAACCTTTACCCTCACCTGTAATTCTCATCGTCACCGCGTGTTGACTACAAGCCAACACATTTGGTTACTTGTGCA | | | | | | | | | |
|  |  |  |  |  |  | GATATTTCTACTTGGTGCCTTGGCAAAACTTGGAGCTACTGTAGTCACCTATCCTCTTCTAGTTGTGAAGGCTCGGCTTC | | | | | | | | | |
|  |  |  |  |  |  | AGGCAAAACAGGTGGTGGCTGGAGACAAAAGACATCAGTACAAAGGCACATTTGATGCCATCATCAAAATGATCCGATAC | | | | | | | | | |
|  |  |  |  |  |  | GAAGGAGTATACGGATTTTACAAAGGGATGGGCACGAAAATCGTCCAGAGTGTTCTGGCTGCTGCAGTCTTGTTCATGGT | | | | | | | | | |
|  |  |  |  |  |  | GAAGGAAGAACTGGTTCGGAGTACTCGATTCCTTCTCACCAAGGGTGCCATTGGCAGAGCAAACTCAAAGCCTCAATGAT | | | | | | | | | |
|  |  |  |  |  |  | TTGATAATGCTTATATGACTCCATTACAGGTCTTTAAGAAGCTATCCAAGGCTGCAATTTCTCTCTACAATCTTTCCTAT | | | | | | | | | |
|  |  |  |  |  |  | TCATCTTAGTGAAGTATTCTTAACCAAACCCTTATTGGAAATTGTTAGGAAATTTGTGTCCT | | | | | | | |  |  |
